# Structural basis for multifunctional roles of human Ints3 C-terminal domain

**DOI:** 10.1074/jbc.RA120.016393

**Published:** 2020-12-03

**Authors:** Jian Li, Xinli Ma, Surajit Banerjee, Sankar Baruah, Nicholas J. Schnicker, Eunmiri Roh, Weiya Ma, Kangdong Liu, Ann M. Bode, Zigang Dong

**Affiliations:** 1 The Hormel Institute, University of Minnesota, Austin, Minnesota, USA; 2 China-US (Henan) Hormel Cancer Institute, Zhengzhou, Henan, China; 3 Northeastern Collaborative Access Team, Cornell University, Advanced Photon Source, Lemont, Illinois, USA; 4 Protein and Crystallography Facility, University of Iowa Carver College of Medicine, Iowa City, Iowa, USA; 5 Department of Cosmetic Science, Kwangju Women’s University, Gwangju, Republic of Korea; 6 College of Medicine, Zhengzhou University, Zhengzhou, Henan, China

**Keywords:** integrator, Ints3, structure, dimer, RNA binding, Ints6 binding, ATM, ataxia-telangiectasia mutated, CD, circular dichroism, CTD, C-terminal domain, DLS, dynamic light scattering, DSBs, double-strand breaks, DTT, dithiothreitol, EMSA, electrophoretic mobility shift assay, HR, homologous recombination, IR, ionizing radiation, PMSF, phenylmethanesulfonyl fluoride, SAD, single-wavelength anomalous diffraction, SLS, static light scattering, UV, ultraviolet

## Abstract

Proper repair of damaged DNA is critical for the maintenance of genome stability. A complex composed of Integrator subunit 3 (Ints3), single-stranded DNA-binding protein 1 (SSB1), and SSB-interacting protein 1 (SSBIP1) is required for efficient homologous recombination-dependent repair of double-strand breaks (DSBs) and ataxia-telangiectasia mutated (ATM)-dependent signaling pathways. It is known that in this complex the Ints3 N-terminal domain scaffolds SSB1 and SSBIP1. However, the molecular basis for the function of the Ints3 C-terminal domain remains unclear. Here, we present the crystal structure of the Ints3 C-terminal domain, uncovering a HEAT-repeat superhelical fold. Using structure and mutation analysis, we show that the C-terminal domain exists as a stable dimer. A basic groove and a cluster of conserved residues on two opposite sides of the dimer bind single-stranded RNA/DNA (ssRNA/ssDNA) and Integrator complex subunit 6 (Ints6), respectively. Dimerization is required for nucleic acid binding, but not for Ints6 binding. Additionally, *in vitro* experiments using HEK 293T cells demonstrate that Ints6 interaction is critical for maintaining SSB1 protein level. Taken together, our findings establish the structural basis of a multifunctional Ints3 C-terminal module, allowing us to propose a novel mode of nucleic acid recognition by helical repeat protein and paving the way for future mechanistic studies.

The human body is constantly challenged by various DNA damage insults, both exogenously from sources such as ionizing radiation (IR), ultraviolet (UV) radiation, and environmental chemicals, and endogenously from sources such as metabolic intermediates, errors in DNA replication, and collapsed replication fork (1, 2). These damages, if not properly repaired, will cause gene mutations, genome instability, and predisposition to cancer and other diseases (3). Coping with these issues, human cells have evolved various pathways to detect, signal, and repair these damages (4).

DNA double-strand breaks (DSBs) are among the most cytotoxic DNA lesions. One of the major mechanisms through which these lesions are repaired is homologous recombination (HR) with a sister chromatid. In the HR pathway, DSBs are recognized by the Mre11-Rad50-Nbs1 (MRN) complex, where it recruits and activates the ataxia-telangiectasia mutated (ATM) protein kinase, a master regulator coordinating repair protein recruitment and checkpoint activation. End resection and Rad51-mediated strand invasion occur in an orderly manner to ensure proper HR-based repair (4, 5, 6). In the DSB repair pathway, single-stranded DNA-binding protein 1 (SSB1, NABP2, SOSSB1) is a central player required for ATM activation and relocates rapidly to DSBs and stimulates strand invasion by RAD51 (7). SSB1 was proposed to directly recruit the Mre11–Rad50–Nbs1 (MRN) complex and stimulate its endonuclease activity (8, 9). Participating in this process, Integrator complex subunit 3 (Ints3, SOSSA) binds SSB1 and SSB-interacting protein 1 (SSBIP1, SOSSC, C9orf80) as a scaffold protein (10, 11, 12, 13). Ints3 also interacts with Integrator complex subunit 6 (Ints6, DDX26a). Depletion of both Ints6 and its paralog DDX26b impairs Rad51 foci formation and homologous recombination repair (14). Mechanistically, Ints3 may regulate the stability and nuclear localization of SSB1 (6, 10, 11, 15). The Ints3–SSB1–SSBIP1 (SOSS1) complex is also shown to stimulate the exo- and endonuclease activities of exonuclease 1 (Exo1) (16).

Ints3 has an N-terminal and a C-terminal domain connected by a linker region (Fig. 1*A*). The structure of the Ints3 N-terminal domain reveals an all α-helical fold that assembles SSB1 and SSBIP1. Among them, SSB1 interacts with Ints3 and ssDNA through two distinct surfaces (15). Ints3 binds ssRNA (30-mer consecutive uracils, rU30), but only very weakly to random ssRNA. For ssDNA (consecutive thymines), Ints3 requires a minimum of 30 nucleotides (dT30) and exhibits lower affinity compared with rU30. Ints3 also binds 30-mer random ssDNA and does not bind dsRNA, dsDNA, or DNA/RNA hybrid (17). Both nucleic acid binding and Ints6 binding properties were attributed to the Ints3 C-terminal domain (14, 17). Apart from this, little is known about the structure and function of this domain. How might it interact with both nucleic acids and Ints6? As its name suggests, Ints3 is one of a 14-subunit integrator complex functioning in small nuclear RNA (snRNA) cleavage and maturation. The well-studied U1, U2, U4, U5, and U6 snRNA are components of the spliceosome, which removes introns from pre-mRNA (18, 19). However, knocking down *Ints3* did not show a clear effect on the processing of several tested snRNA either in *Drosophila* or in human cells (20, 21). Still a role for Ints3 in snRNA biogenesis could not be excluded and awaits careful exploration.

**Figure 1:**
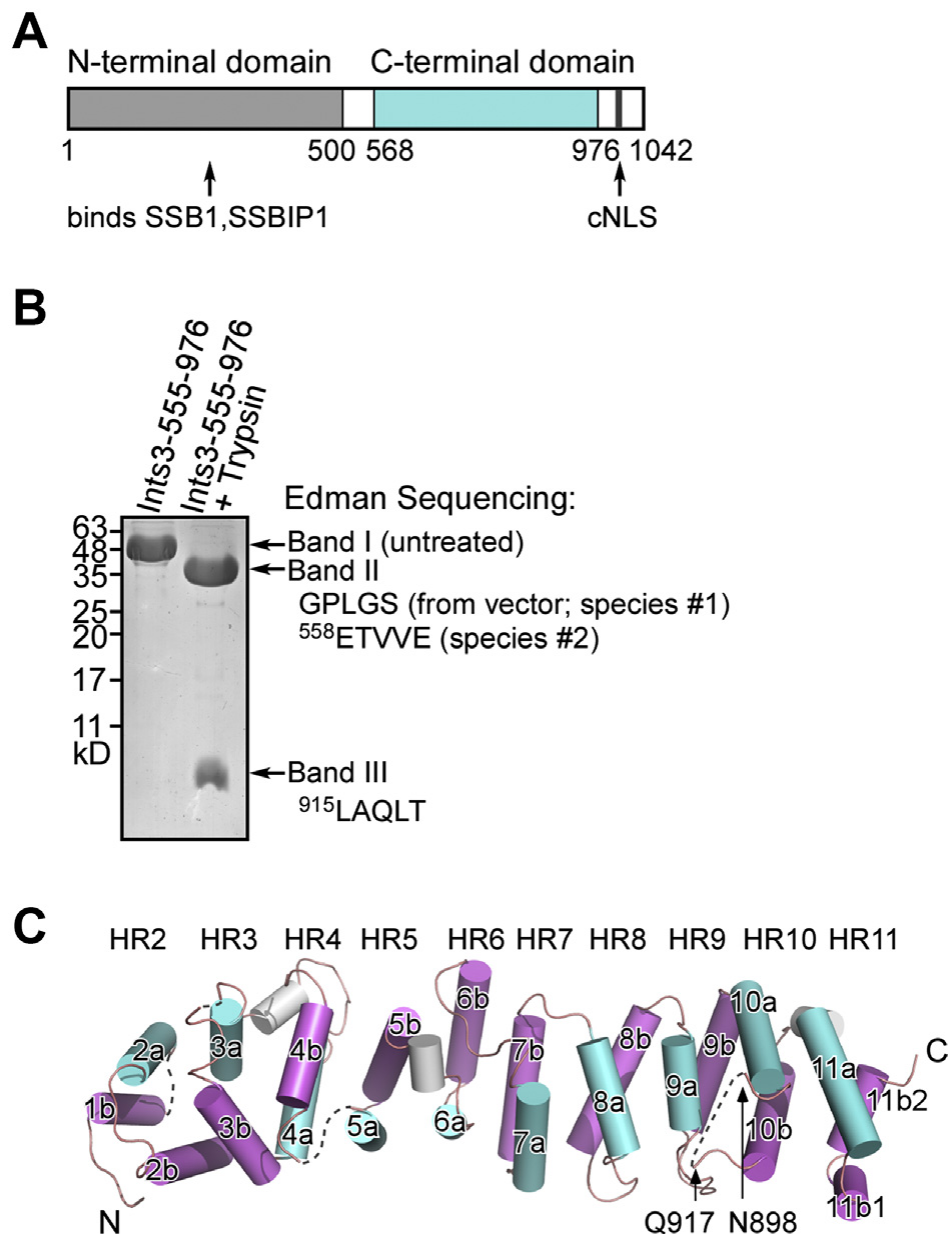
**Overall structure of the human Ints3 C-terminal domain.***A*, domain organization of human Ints3. Ints3 contains an N-terminal SSB1/SSB1P1 binding domain (residues 1–500, PDB ID 4OWT), a C-terminal domain (residues 555–976) followed by a classical importin α/β pathway-dependent nuclear localization signal (cNLS), predicted by cNLS mapper (55). *B*, limited trypsin treatment generates two bands, Band II and III, from full-length C-terminal domain (Band I). N-terminal amino acid sequences of band II and III were obtained by Edman degradation method. *C*, the Ints3 C-terminal domain is an elongated HEAT-repeat α-solenoid consisting of 11 HEAT repeats (HR1-11). Each repeat is composed of an a-helix (*cyan*) and a b-helix (*magenta*), connected by a short linker. Helix 1a of HR1 was not modeled due to weak density. One confirmed trypsin cleavage site (between K914 and L915) lies within the HR10 intrarepeat loop where electron density for residues N899–A916 is missing.

In this study, we determined the crystal structure of the human Ints3 C-terminal domain. The structure reveals a previously unknown HEAT-repeat superhelical fold. Structural and biochemical studies demonstrate that the Ints3 C-terminal domain forms a dimer and an extended basic groove formed upon dimerization is involved in ssRNA/ssDNA binding, representing a novel mode of nucleic acid recognition. On the opposite side of this groove, a cluster of highly conserved residues is critical for Ints6 binding. Our studies thus identified a dimeric multifunctional module in the Ints3 C-terminal domain.

## Results

### *In situ* proteolysis for the crystallization of the human Ints3 C-terminal domain

To understand the function of human Ints3 C-terminal domain, we used X-ray crystallography to determine its structure. Based on secondary structure predictions from PSIPRED (22), Phyre2 (23), and I-Tasser (24) (Fig. S1), one initial construct, Ints3-555-976, was designed and screened for crystal growth. Although several hits were obtained, they were not consistently repeatable. Limited trypsin digestion revealed a stable band (Band II) slightly shorter than the untreated protein (Band I, Fig. S2*A*). Western blotting against the N-terminal His-tag showed this band II to be the N-terminal portion of the expressed construct (Fig. S2*B*). A new construct, Ints3-555-899, was used for crystallization studies, which has approximately the same size as the trypsin treatment-generated stable fragment in SDS-PAGE gel (Fig. S2*C*). This new construct, however, also did not provide reproducible crystals.

Trypsin treatment actually generated two bands (Band II and III) from the full-length protein (Band I), which was revealed by running a high-percentage acrylamide gel. Band II corresponds approximately to construct Ints3-555-899, while Band III is smaller than an 81-amino-acid construct Ints6-807-887 (Fig. 1*B* and Fig. S2*C*). Given that a disordered 20-amino-acid loop rich in Arg, Lys, and Ser exists after residue 899 (Fig. S1), we hypothesize that trypsin cleaves this internal loop and the two parts still bind tightly to each other. Band III, predicted to be roughly residues 920–976, is indeed less than 81 amino acids. Protein N-terminal sequencing by Edman degradation further supports our hypothesis. Band II contains two species. One is composed of amino acids derived from the pGEX-6p-1 vector. The other species starts from residue 558, which is C-terminal to K557. Band III starts from residue 915, which is after K914 of the internal loop (Fig. 1*B* and Fig. S1). These cleavage sites are consistent with the specificity of trypsin.

Finally, *in situ* proteolysis was used to screen crystals (25, 26), by adding a trace amount of trypsin in the protein sample (1:1000 w/w). This gave robustly reproducible and diffraction-quality crystals.

### Overall structure of the human Ints3 C-terminal domain

The structure of Ints3-555-976 was solved by single-wavelength anomalous diffraction (SAD) and data sets from four different selenomethionine-substituted crystals were merged to facilitate substructure determination and phase calculation (27) (Methods and Table S1; see Fig. S3 for structure validity and map quality). The structure of the human Ints3 C-terminal domain reveals an elongated, HEAT-repeat-like, all α-helical structure (Fig. 1*C*). The monomer has an approximate dimension of 100 x 30 x 25 Å. Electron density map and secondary structure predictions from multiple sources are consistent with the total number of prominent helices, which could be assigned to 11 HEAT repeats (HR1-11). Each repeat is composed of an antiparallel α-helical pair or zigzag, referred to as a- and b-helices (Fig. S3*B*). The adjacent repeats pack roughly parallel to each other, stacking into an elongated array. Owing to twists between neighboring repeats, the a- and b-helices form a right-handed α-solenoid (Fig. 1*C*). Due to weak density as a result of disorder, helix 1a of the HR1 could not be modeled. HR5-11 shows the best map quality (Fig. S3*B*), which is the region that harbors functionally critical residues and the focus of this manuscript. Because of the degenerate consensus sequence (28), the existence of HEAT repeats in the Ints3 C-terminal domain was not recognized previously. One well-conserved characteristic is that the a- and b-helices are amphiphilic. Hydrophobic residues face inward to form the hydrophobic core, whereas the hydrophilic residues are exposed. Aspartic acid residues are often found in the turn region (Fig. S3*C*).

The Dali server was used to search for structural homologues (29). Surprisingly, the first two hits are RNA polymerase II phosphorylated C-terminal domain (phospho-CTD) motif interacting domain (CID; PDB ID 5LVF, 4FLB, Z score around 8.9, rmsd around 2.6 Å) (Fig. S4*A*). However, in our experiment, full-length Ints3 did not show RNA polymerase II binding activity comparable with that of the well-established RPRD1b (30) (Fig. S4*B*). Despite overall structural similarity, the local conformations around the CTD binding pocket are different. Most of the top hits are HEAT-repeat proteins, including the TOG (tumor overexpressed gene) domain from the Zyg9 protein (PDB ID 2OF3, Z score 6.4, rmsd 3.8 Å, for 168 structurally aligned residues). TOG is a well-characterized domain containing 6 HEAT repeats, and the structurally aligned region roughly covers HR5-10 of the Ints3 C-terminal domain. This analysis increases our confidence that the Ints3 C-terminal domain is indeed a HEAT-repeat protein.

### Ints3 has a dimeric C-terminal domain

**Figure 2:**
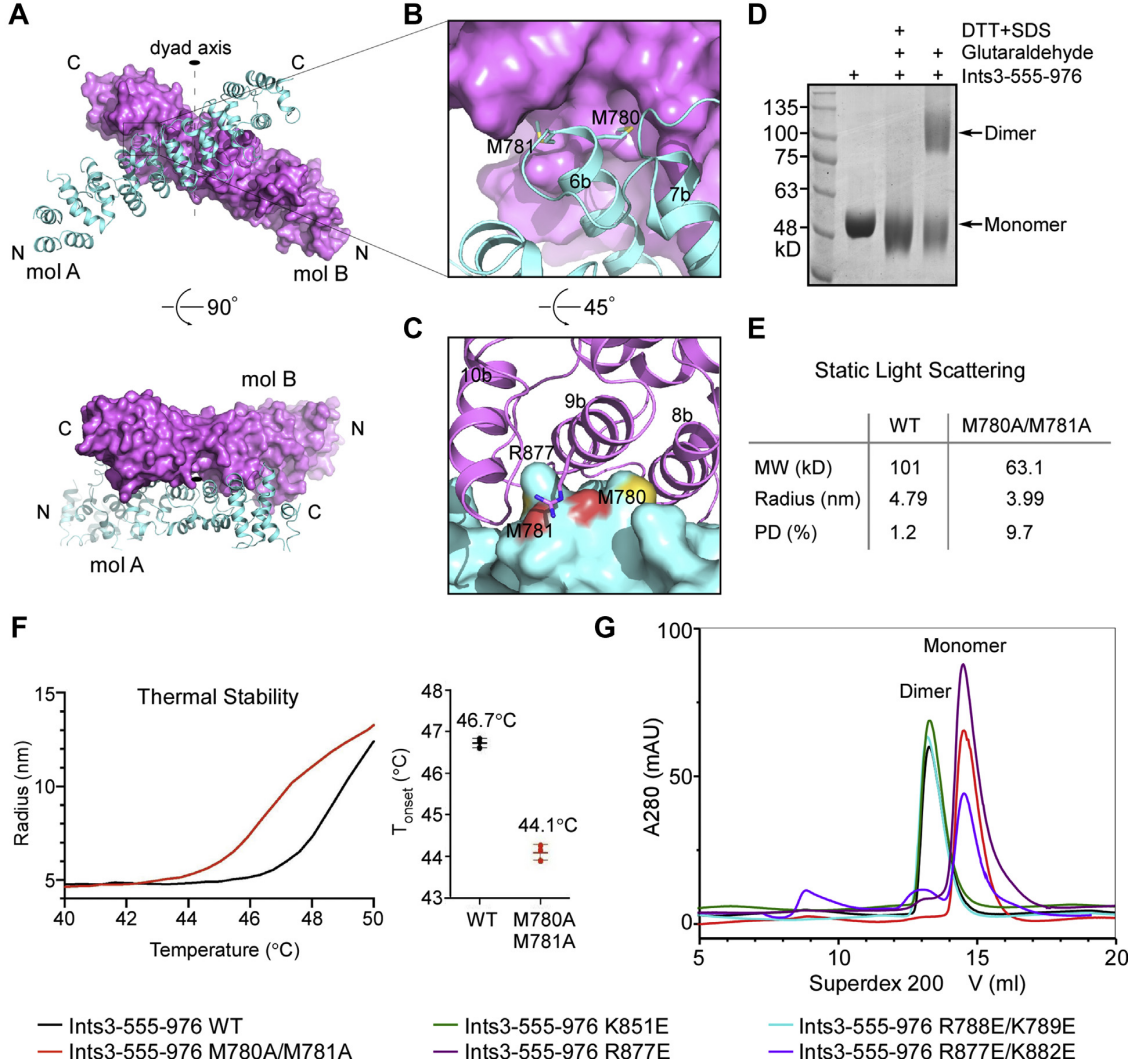
**The Ints3 C-terminal domain is a dimer.***A*, analysis of the crystal packing by using PISA (31) suggested a stable dimeric split. The two molecules are related by a twofold rotational symmetry and the dyad axis is shown as a black oval. Molecule A (mol A) in cyan is shown as a cartoon, and molecule B (mol B) in *magenta* is shown in surface style. *B*, closeup view of an experimentally verified contact site. M780/M781 side chains from helix 6b of mol A reach into two adjacent pockets on the surface of mol B. *C*, similar view as in (*B*), but turned 45° to better show details. Molecule A is in surface representation and mol B is shown as a cartoon. Helix 9b of mol B sits in a saddle formed by M780/M781 of mol A. M780/M781 side chain sulfur atoms are colored *orange* and carbonyl oxygen atoms are in *light red*. Positively charged R877 side chain from mol B helix 9b is in close proximity to mol A M780/M781 carbonyl O atoms. *D*, chemical cross-linking using glutaraldehyde (final concentration 0.075%) reveals a dimer band in SDS-PAGE gel. For negative control, 1% SDS and 20 mM DTT were added before the cross-linking reaction. *E*, molecular weights determined by static light scattering are 101 kD and 63.1 kD for the wild-type and M780 A/M781 A mutant protein, respectively. This suggests that M780 A/M781 A double mutation has largely converted the dimeric Ints3 C-terminal domain into a monomer. *F*, M780 A/M781 A mutant has reduced thermal stability compared with wild-type protein, as shown by the dynamic light scattering measured aggregation on-set temperature (T_onset_). *G*, gel filtration profile of wild-type and mutant Ints3 C-terminal domain obtained from a Superdex 200 Increase 10/300 Gl column. These proteins elute with two distinct peaks, one corresponding to dimer and the other corresponding to monomer. M780 A/M781 A and R877E-containing mutation disrupt dimer formation, consistent with structural analysis in (*B, C*).

Two copies of the molecule are found in the crystallographic asymmetric unit. PDBePISA (Proteins, Interfaces, Structures, and Assemblies) is a powerful tool for the analysis of macromolecular interfaces (31). Analysis by PISA suggested that a 1702.3 Å^2^ dimeric surface might be stable and present in solution. An X-shaped dimer would form utilizing this interface (Fig. 2*A*). Because of the twofold rotational symmetry, helix 6b from molecule A stretches out slightly and sits on top of helices 8b/9b from molecule B, and vice versa (Fig. S5*A*). The interface is composed of 21 hydrogen bonds, three salt bridges, and numerous hydrophobic interactions. Of these, each of two hydrophobic residues, M780/M781 of helix 6b, sticks into a cavity on the surface of the other molecule (Fig. 2*B*). Meanwhile, R877 side chain of helix 9b from the second molecule is in close proximity to the carbonyl oxygen atoms of M780/M781 (3.6 Å, Fig. 2*C*). For those three residues, side chain electron density is well defined (Fig. S5, *B*–*C*).

Evidence from multiple sources supports that the Ints3 C-terminal domain is a dimer. First, chemical cross-linking using glutaraldehyde revealed a dimer band in SDS-PAGE gel (Fig. 2*D*). Second, by static light scattering, the molecular weight of the wild-type protein was determined to be 101 kD, corresponding to a dimer (calculated Mw from the sequence is 50.3 kD). The molecular weight of the M780 A/M781 A mutant was measured to be 63.1 kD, suggesting that these mutations have disrupted dimer formation (Fig. 2*E*). In addition, compared with dimeric wild-type protein, monomeric M780 A/M781 A mutant had significantly reduced thermal stability, as revealed by the onset-of-melting temperatures (T_onset_) (Fig. 2*F*). Third, wild-type and five mutants used in this study were characterized by carefully controlled gel filtration experiments. Wild-type, K851 E, and R788 E/K789 E eluted at a dimer peak, while M780 A/M781 A, R877 E, and R877 E/K882 E all eluted at a monomer peak. The two peaks are distinct and well separated (Fig. 2*G*). Finally, the monomeric mutants still retain a low percentage of dimeric species (the small bump preceding the major peak in Fig. 2*G*), which appeared to increase as the protein sample gets old. For M780 A/M781 A mutant, a minor 92 kD peak and a major 56 kD peak were resolved using an independently calibrated gel filtration column (Fig. S5*D*). Collectively, these data suggest that M780, M781, and R877 are critical for a dimeric Ints3 C-terminal domain.

### A basic groove formed upon dimerization binds single-stranded RNA and DNA (ssRNA/ssDNA)

**Figure 3:**
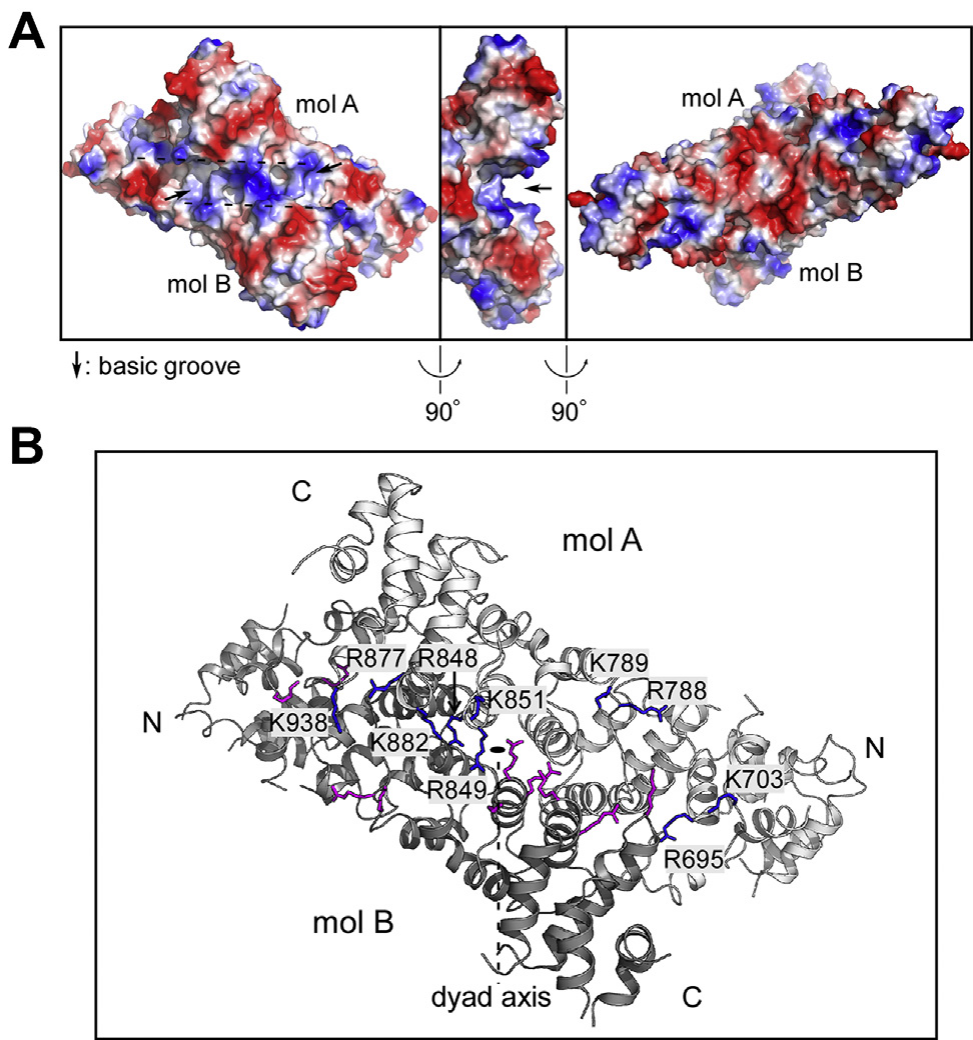
**A basic groove identified on the dimer surface.***A*, analysis of surface charge distribution revealed a deep and extended basic groove sitting on top of the dimeric interface, possibly involved in ssRNA binding (*left and middle panels, arrows*). The opposite side is largely negatively charged (*right panel*). *B*, this basic groove is lined with a series of positively charged Arg and Lys residues, contributed from both molecules (*colored blue* and *purple*, respectively) and shown in stick representation. Only those residues from molecule A are labeled with name and position in the sequence. Molecules A and B are colored *light gray* and *dark gray*, respectively.

The Ints3 C-terminal domain has ssRNA and ssDNA binding activity (17). Protein–RNA interfaces prefer positively charged residues (32). Electrostatic surface analysis of the C-terminal domain dimer revealed an extended groove with a strong positively charged potential. The opposite side is highly negatively charged (Fig. 3*A*). This groove is created upon dimerization of the two molecules and sits on top of M780/M781, two residues critical for dimer formation. A series of basic residues (Arg and Lys) form the lining wall of this groove, including a center-positioned K851, R788/K789, R877 (important for dimerization), and others. Deep pockets could be found at the bottom of the groove, which might accommodate the RNA bases (Fig. 3*B*). We propose that the extended groove is the ssRNA/ssDNA binding site, and mutations disrupting dimer formation will also affect nucleic acid binding.

**Figure 4:**
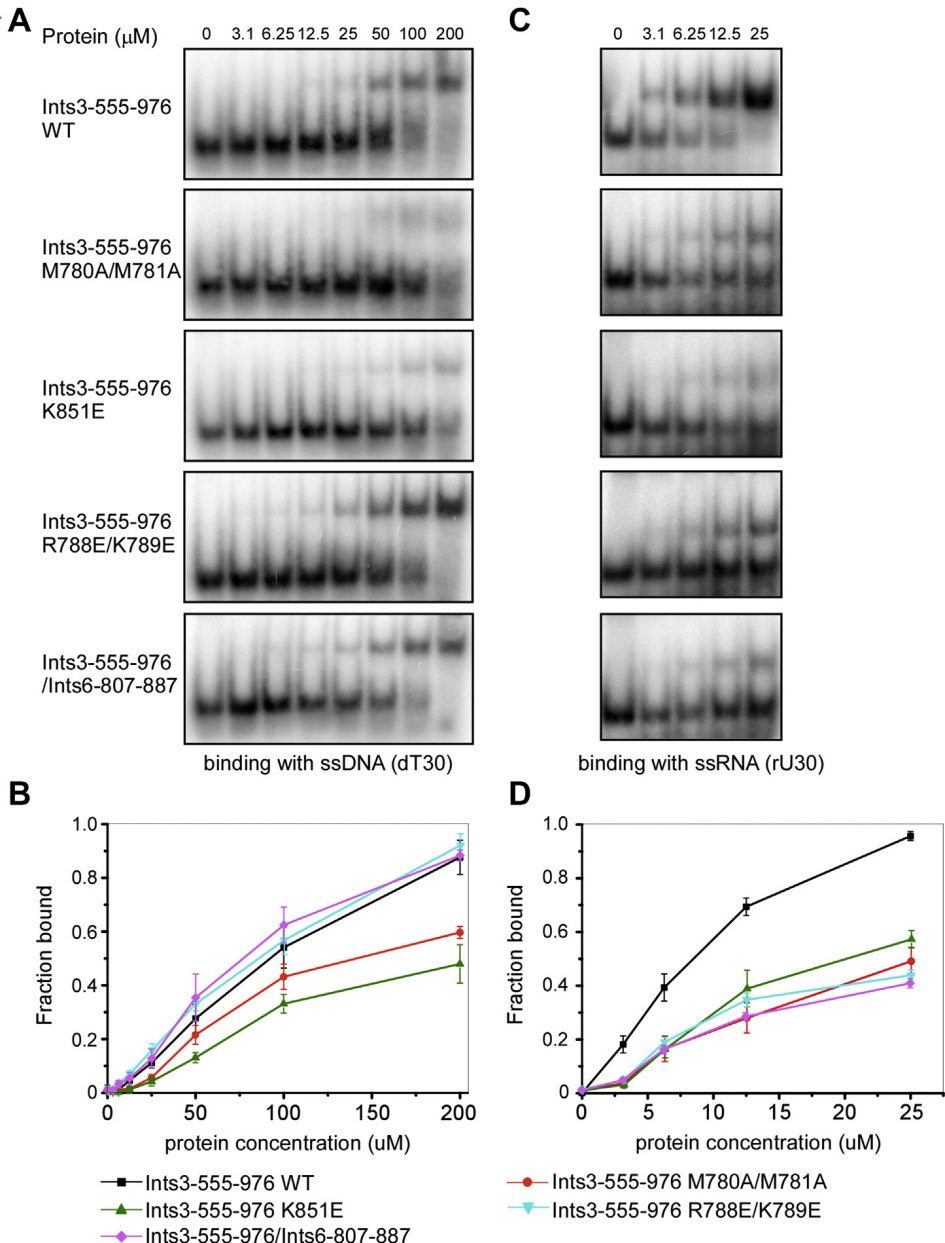
**A basic groove formed upon dimerization and centered on K851 is responsible for single-stranded DNA/RNA binding.***A*, binding of the Ints3 C-terminal domain wild-type, mutant, and Ints6-807-887 complex protein with single-stranded DNA (30 mer consecutive thymines, dT30). The protein concentrations used were up to 200 μM. Representative images of EMSA are shown. *B*, quantification of (*A*) from three independent repeats. Error bars represent standard deviation. *C*, binding of the Ints3 C-terminal domain wild-type, mutant, and Ints6-807-887 complex protein with single-stranded RNA (30 mer consecutive uracils, rU30). The protein concentrations used were from 0 to 25 μM. Representative images of EMSA are shown. *D*, quantification of (*C*) from three independent repeats. Error bars represent standard deviation.

To directly test this hypothesis, relevant mutant proteins were purified to homogeneity (Fig. S6*A*). Electrophoretic mobility shift assay (EMSA) results showed that Ints3 C-terminal domain binds ssRNA (30-mer consecutive uracils, rU30) much more tightly than ssDNA (30-mer consecutive thymines, dT30). At the protein concentration of 25 μM, roughly 100% binding to rU30 was achieved, while for dT30, the shifted species percentage was rather low (Fig. 4, *A* and *C*). Supporting our hypothesis, the monomeric mutant M780 A/M781 A and the K851 E charge mutant had significantly reduced binding capacity toward both rU30 and dT30. R788 E/K789 E mutant only showed reduced binding to rU30, but not to dT30 (Fig. 4 and Fig. S6, *B–E*). K851 E and R788 E/K789 E mutants still form dimers, excluding the possibility that the mutation acts by disrupting the dimer (Fig. 2, *G*). Overall, we conclude that a basic groove formed upon dimerization and centered on K851 is involved in ssRNA/ssDNA binding.

Unexpectedly, once complexed with an Ints6 fragment (Ints6-807-887), the Ints3 C-terminal domain had compromised binding to rU30, but not to dT30, suggesting a potential regulatory mechanism (see discussion) (Fig. 4 and Fig. S6, *D–E*).

### A conserved surface patch is critical for Ints6 interaction

The Ints3 C-terminal portion interacts with Ints6, and Ints6-747-887 was implicated in this interaction (14). Ints6 residues 747–806 are neither conserved nor structured (Fig. S7, *A*–*B*). Thus, Ints6-807-887 was selected to reconstitute complex with the Ints3 C-terminal domain by coexpression method. The Ints6-807-887 region contains two predicted α-helices and is able to form a complex with Ints3. Shorter fragments having one of the helices failed to interact with Ints3 (Fig. S7, *B*–*C*). For the Ints3 part, we first demonstrated that both Ints3-555-976 (HR1-11) and Ints3-555-899 (HR1-10a) are able to form stable complexes with Ints6-807-887 when coexpressed and copurified from *Escherichia coli* BL21 (Fig. S8*A*). HEAT repeats from either the N- or C-terminus of the Ints3 C-terminal domain were systematically deleted, and the Ints6-interacting region was mapped roughly to HR6-8 (Fig. S8 and Fig. S9).

**Figure 5:**
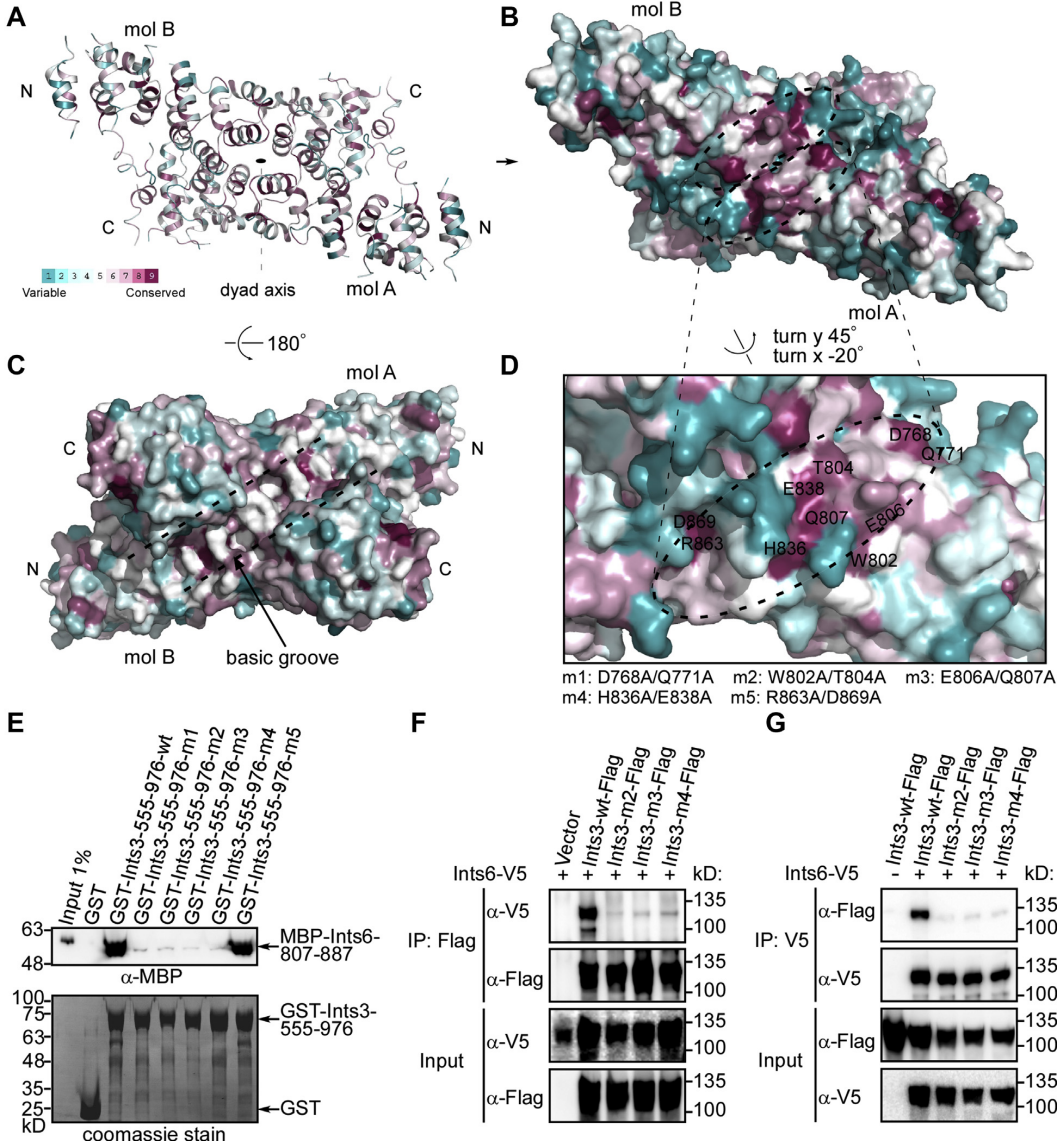
**A cluster of conserved residues is critical for Ints6 binding.***A*, Cartoon and (*B*) surface representations showing the distribution of conserved residues from ConSurf analysis (33). Each molecule contributes a stretch of conserved residues (*dotted oval*), and two such stretches are close together forming a larger patch. *C*, on the opposite side of this conserved patch is the basic groove proposed to bind ssRNA, which is moderately conserved. *D*, enlarged view of the conserved surface from molecule A. Positions of the conserved residues and mutations used in this study are indicated. Of these, R863/D869, later found to be dispensable for Ints6 binding, is slightly separated from the other more congregated ones. *E*, pull-down of Ints6-807-887 by GST-tagged Ints3 C-terminal domain mutants (m1-m5). GST-tagged Ints3 was first immobilized on glutathione Sepharose beads and then incubated with a purified MBP-tagged Ints6-807-887 protein. After extensive washing, MBP-Ints6-807-887 bound to Ints3 was detected using an MBP antibody. Representative result from at least three repeats is shown. *F*, Coimmunoprecipitation between full-length Flag-tagged wild-type or mutant Ints3 and V5-tagged Ints6. Immunoprecipitations (IPs) were performed with anti-Flag M2-agarose beads, and precipitated proteins were detected with a V5 antibody. Representative result from at least three repeats is shown. *G*, similar as in (*F*), but IPs were performed with anti-V5 agarose beads, and precipitated proteins were detected with a Flag antibody.

Conserved surface residues represent functionally critical regions of a protein. We hypothesize that the most conserved residues in the Ints3 C-terminal domain are involved in Ints6 binding. Through Consurf server analysis (33), one highly conserved surface area was identified (Fig. 5, *A*–*B*). Each monomer displays a stretch of conserved residues (dashed oval), and owing to the close proximity to the symmetry axis, such two stretches are juxtaposed (Fig. 5*B*). This patch is on the opposite side of the basic groove identified above, which is moderately conserved (Fig. 5*C*). For each molecule, the conserved stretch is composed of residues D768, Q771, W802, T804, E806, Q807, H836, E838, R863, and D869. The last two residues appear more isolated from the rest (Fig. 5*D*). D768/Q771 are located in the intrarepeat turn (linker) region within HR6, W802/T804/E806/Q807 within HR7, H836/E838 within HR8, and R863/D869 within HR9. We assume that these residues are involved in the Ints6 interaction. To test this idea, five mutants were made, and the Ints6 binding ability was assessed through coexpression and reverse pull-down. Of these, the Ints3-555-976 D768 A/Q771 A (mutant 1, m1) and R863 A/D869 A (m5) retained the ability to interact and stabilize Ints6-807-887 when coexpressed (Fig. S10*A*). GST-tagged Ints3-555-976 W802 A/T804 A (m2), E806 A/Q807 A (m3), H836 A/E838 A (m4) all failed to pull down MBP-Ints6-807-887. *In vitro* GST pull-down was also performed to validate these findings. In this experimental setting, Ints3-555-976 mutants m1, m2, m3, and m4 all had greatly reduced binding with Ints6-807-887 (Fig. 5*E*). R863/D869 (corresponding mutant m5) is dispensable, while W802/T804, E806/Q807, H836/E838 (corresponding mutants m2, m3, m4) are absolutely required for Ints6 binding. This is consistent with the mapped interacting region (HR6-8) and also in line with the fact that of the conserved patch residues, R863/D869 appears isolated from the other more congregated residues. The inconsistency of m1 mutant may be because coexpression is a condition more favorable for complex formation than *in vitro* pulldown.

Unlike the ssRNA binding ability, a dimeric Ints3 C-terminal domain is not required for the Ints6 interaction. All of the M780 A/M781 A, R877 E/K882 E, and R877 E mutant proteins are able to form a complex with Ints6-807-887, whereas E806 A/Q807 A (m3) had the most adverse effects, as shown by the coexpression and reverse pull-down assays (Fig. S10*B*).

To examine the effect of these mutations (m2, m3, m4) in the context of the full-length protein, a coimmunoprecipitation assay was performed by transfecting HEK 293T cells with Flag-tagged Ints3 and V5-tagged Ints6 full-length constructs. Results showed that these mutations significantly affected Ints6 binding (Fig. 5, *F*–*G*), similar as in the case of using a partial domain and fragment. Collectively, our data showed that a cluster of conserved surface-exposed residues, W802/T804, E806/Q807, and H836/E838, are critical for Ints6 binding.

### Ints6 interaction is critical for maintaining SSB1 protein level

**Figure 6:**
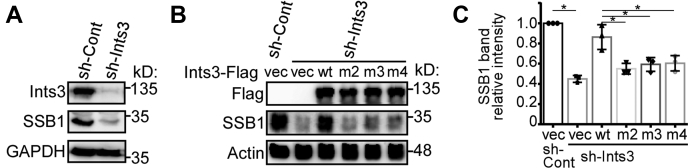
**Ints6 interaction is critical for maintaining SSB1 protein level.***A*, knocking down Ints3 in HEK 293T cells reduces SSB1 protein level. *B*, in knockdown cells, SSB1 level could be rescued by wild-type Ints3, but not mutants with defect in Ints6 binding. A representative image of western blot is shown. *C*, quantification of the SSB1 band intensity in (*B*) obtained from three independent repeats. Error bars represent standard deviation. Significance was tested by unpaired Student’s *t*-test: ∗, *p* < 0.05.

One important mechanism of how Ints3 exerts its function in the DSB repair pathway is by regulating the SSB1 protein level (10, 11, 15). We confirmed that knocking down Ints3 in HEK 293T cells reduces SSB1 protein abundance (Fig. 6*A*). Re-expression of wild-type Ints3 could restore SSB1 protein level, but Ints3 mutants with a defect in Ints6 binding failed to rescue (Fig. 6, *B*–*C*). This is consistent with a role of Ints6 in the DSB repair (14). For other types of Ints3 mutants including the dimerization mutants and the ssRNA/ssDNA binding mutants, we could not obtain consistent results in this assay.

## Discussion

In this study, we solved the crystal structure of the human Ints3 C-terminal domain and performed detailed biochemical characterization making use of more than ten mutants. For all the proteins purified by gel filtration, peak positions were as expected, suggesting that the proteins were well folded. Three representative mutants were tested directly by circular dichroism (CD) and showed α-helical secondary structure consistent with wild-type Ints3 (Fig. S11).

**Figure 7:**
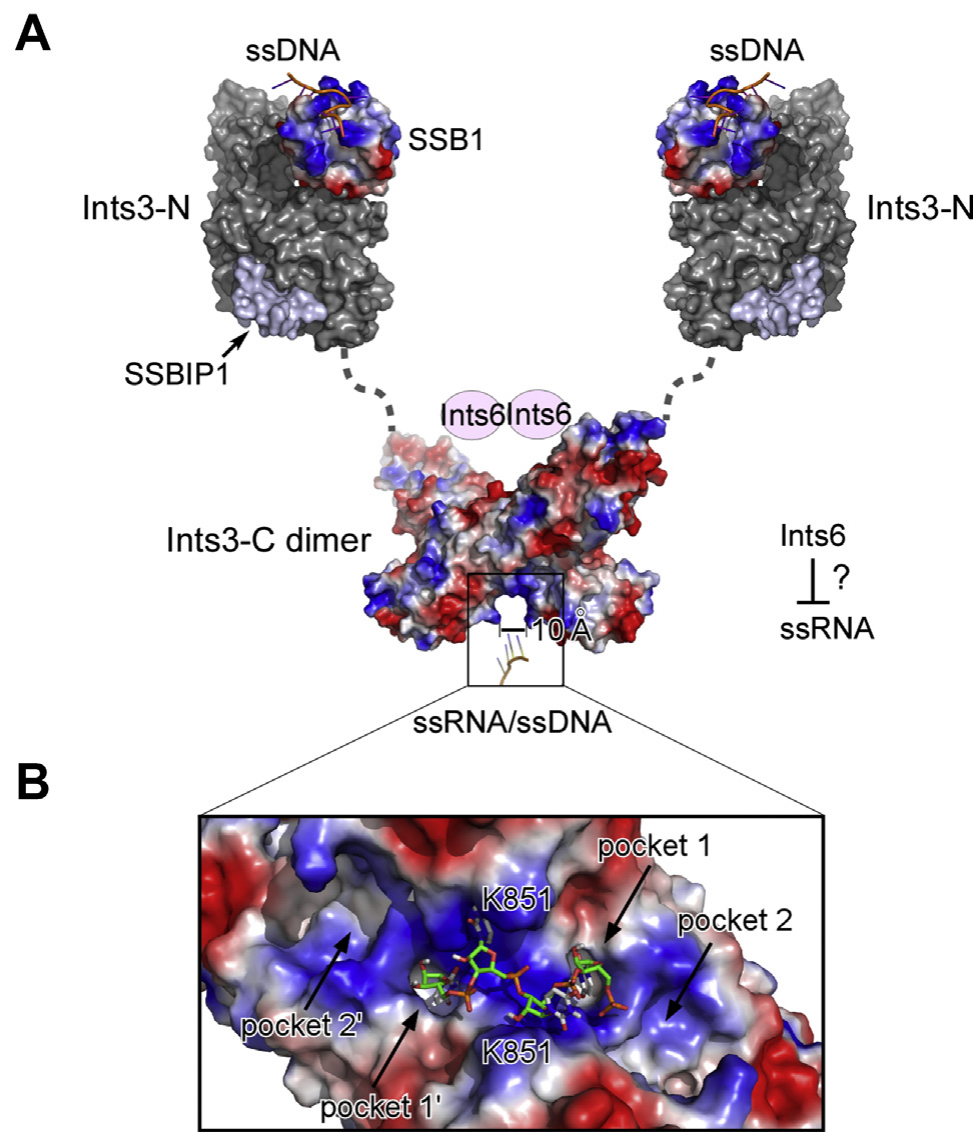
**Composite model of full-length Ints3 in the DSB repair pathway.***A*, Ints3 N-terminal domain-SSB1-SSBIP1 is based on PDB 4OWX, 4OWT. An Ints3 C-terminal domain dimer is reported here. Through dimerization, the whole complex will have three nucleic acid binding epitopes, two from SSB1 and one from Ints3 C-terminal domain. Ints6 and ssRNA are proposed to occupy the opposite side of the dimer. *B*, AutoDock Vina (44) generated one top model of the Ints3 bind with a 4-nucleotide RNA (rU4, UUUU) with a reported affinity of –7.9 kcal/mol. The basic groove is suitable for ssRNA binding, and four pockets in the groove potentially accommodate the RNA nucleobases.

The C-terminal domain exists as a dimer (Fig. 2). Given that the N-terminal and C-terminal domains are connected by an approximate 70-amino-acid linker, we propose that full-length Ints3 also exists as a dimer in cells. Indeed, fractionation of HeLa or 293T nuclear extracts by gel filtration revealed that the Ints3-SSB1-SSBIP1 complex elutes in fractions between 440 and 670 kD, implying more than one copy of each subunit (8, 11). This dimeric structure might be important for the Ints3 function in the DSB repair pathway. Ints3 interacts with SSB1 through the N-terminal domain. A dimeric Ints3 C-terminal domain would bridge two SSB1 molecules in close proximity (Fig. 7*A*). Similar to what was shown for many nucleic acid binding modules (34, 35, 36), having two SSB1 in a row allows higher ssDNA binding affinity by adding up the total binding area and by increasing the local concentration of the second binding module. Thus, cooperative binding could happen. Even more complicated function might be achieved, such as sliding or inchworm movement by transiently release one SSB1 at a time from the ssDNA substrate. Another obvious consequence is that the bridged two SSB1 could bind a much longer stretch of ssDNA, and therefore, the sequence/structural specificity toward the DNA substrate could change compared with a single SSB1. Two different molecules of ssDNA could also be bound to, fulfilling a tethering function in this scenario. In addition to this, the Ints3 C-terminal domain also has its own ssDNA binding ability, albeit much lower compared with its ssRNA binding activity. Both the ssDNA and ssRNA binding activity could be important in the DSB repair pathway. Even though the mechanism is unclear, the roles of RNA in DSB repair are rapidly emerging and a number of well-documented DNA repair factors have been described to have RNA-binding capability (37). Overall, the dimeric Ints3 makes the Ints3–SSB1–SSBIP1 complex more like a conventional RPA protein having multiple ssDNA binding OB-fold/epitope (Fig. 7*A*), and the use of multiple homologous domains working together to bind ssDNA is a nearly ubiquitous feature (6, 36). Our composite model could explain why the Ints3–SSB1–SSBIP1 complex shows a 30-fold higher affinity for ssDNA than SSB1 alone (16). This dimeric configuration is also reminiscent of multiple dimeric proteins function in the DSB repair pathway, most importantly the MRE11–RAD50–NBS1 (MRN) complex (38), ATM, and ATR (39). Both SSB1 and Ints3 have been reported to interact directly with NBS1 (8, 10), and the whole MRN–Ints3–SSB1 machinery could be a dimer.

A previous report for the first time studied the nucleic acid binding property of human Ints3 (17). Based on our structural and mutational studies, we confirmed that the Ints3 C-terminal domain is a bona fide ssRNA/ssDNA binding module (Fig. 3 and Fig. 4). Helical-repeat proteins are known to be able to bind nucleic acids. The maize chloroplast Pentatricopeptide-repeat (PPR) protein PPR10 forms a right-handed superhelical spiral and uses the inner helices to bind ssRNA (40). Pumilio/FBF (PUF)-repeat proteins stack into a superhelical crescent and also use the concave inner helices to bind ssRNA (41, 42). Compared with PPR and PUF proteins, the Ints3 C-terminal domain only has a slight curvature and no positively charged patch exists on the surface of inner helices. Instead, we found that a deep basic cleft formed upon dimerization most likely to be the ssRNA/ssDNA binding site (Fig. 3 and Fig. 4). Recognition of RNA through a dimeric protein interface could only be found in a few examples (34, 43). It is as expected that Ints3 as a subunit of the integrator complex involved in snRNA processing binds rU30 since major types of snRNA (U1, U2, U4, U5, U6) all contain high uridine content. The narrowest part (also the center part) of the groove, where the extremely important K851 is situated as a bottleneck, has a diameter between 10 and 15 Å (Fig. 7*A*). This is significantly narrower than dsDNA and dsRNA (between 20–26 Å), which explains why Ints3 does not bind these double-stranded nucleic acids. Furthermore, we performed preliminary RNA–protein docking using the automated AutoDock Vina program (44). Figure 7*B* shows one binding pose of the 4-nucleotide RNA (rU4, UUUU) with a reported affinity of –7.9 kcal/mol. Four pockets in the groove could be identified and were named pockets 1, 1’, 2, 2’ due to the symmetry of the dimer. These pockets frequently accept the nucleobases in our docking analysis with various ligands (rU4, rU3, rU2, AMP, UMP). Pockets 1 and 1’ are able to accommodate both uracil and larger adenine bases. These pockets and the rotational symmetrical nature of the C-terminal domain could determine its sequence and structural specificity toward ssRNA, if ever exists in cells. Based on the dimension of this groove and the contour length of each nucleotide (∼0.5 nm) (45), a 26-nt ssRNA might best fit this channel. Together, we propose a novel mode of ssRNA recognition by a HEAT-repeat domain through the dimeric interface.

Compared with ssDNA, ssRNA appears to be a better substrate of the Ints3 C-terminal domain. Knocking down *Ints*3 did not show a clear effect on the processing of U1, U2, U4, U6 snRNA in *Drosophila* S2 cells and on the processing of U2 snRNA in HeLa cells (20, 21). However, the Ints3 C-terminal domain as an ssRNA binding module might be involved in the processing of other types of snRNA, or it may participate in steps that do not result in failure of snRNA 3’-end cleavage. For both ssRNA and ssDNA, monomeric mutant and K851 E mutant have dramatically reduced binding. R788 E/K789 E mutant and Ints6 complex only shows reduced binding to ssRNA (Fig. 4). One possibility is that ssRNA occupies both the center and periphery of the binding groove and shows higher affinity, whereas ssDNA as a suboptimal substrate only occupies the center of the groove. Therefore, because R788/K789 and presumably a portion of the Ints6 binding area (see below) are relatively peripheral, these proteins have not much change on their ssDNA binding ability. The Ints3-555-976/Ints6-807-887 has reduced binding to ssRNA, suggesting that Ints6 might regulate the access of ssRNA to the Ints3. We could not directly test the competition relation because Ints6 expressed poorly without its binding partner.

We also identified a cluster of conserved residues, on the opposite side of the basic groove, which is critical for the Ints6 interaction (Fig. 5). The Ints3 binding region in Ints6 is predicted to have two helices with a total of 80 amino acids. This implies that the Ints6 binding region on the Ints3 C-terminal domain may not be confined to this small cluster of critical amino acids. Indeed, although Ints3-555-899 formed a stable complex with Ints6-807-887, results from *in vitro* GST pull-down indicate that Ints3-555-899 had reduced binding compared with Ints3-555-976 (Fig. S8*B*). Thus, despite that the key areas of Ints6 and ssRNA binding situate on the opposite side of the dimer, Ints6 binding might also involve a peripheral area (residues 900–976) that could interfere with ssRNA binding. Unlike the nucleic acid binding, dimerization is not required for Ints6 binding (Fig. S10*B*). This suggests that Ints3 C-terminal domain dimer might bind two molecules of Ints6-807-887 (2:2 complex). Each monomer has one very conserved patch for Ints6 binding. Although they are adjacent, each monomer (patch) would bind one molecule of Ints6. Cocrystal structures of the Ints3 C-terminal domain with ssRNA or Ints6 will be needed to elucidate the exact binding modes.

Altogether, we found the Ints3 C-terminal domain as a dimeric multifunctional module. Structural and biochemical analysis lay the groundwork for future insightful mechanistic studies in the cellular context.

## Experimental procedures

### Protein expression and purification

The human *Ints3* gene was purchased from Mammalian Gene Collection (MGC; Dharmacon, Inc; Lafayette, CO, USA), while *Ints6* was from DNAsu (Tempe, AZ, USA). The Ints3 sequence corresponds to UniProtKB Q68E01-2 with 1042 amino acids in its full-length form. For expression, all GST-tagged constructs were cloned into pGEX-6p-1 (GE Healthcare; Chicago, IL, USA), and all His-tagged constructs were cloned into pRSFDuet-1 (Novagen; Madison, WI, USA) vector. All constructs were verified by sequencing (Integrated DNA Technologies, IDT; Coralville, IA, USA).

For structural studies, Ints3-555-976 was expressed as an N-terminal GST-tagged protein in the *E. coli* strain BL21-CodonPlus (DE3)-RIPL (Agilent; Santa Clara, CA, USA). Cells were cultured in Lysogeny broth (LB medium) with 100 μg/ml ampicillin at 37 °C until the OD_600_ of the culture reached 0.8–1.0. Protein expression was induced by 0.25 mM isopropyl-β-D-thiogalactopyranoside (IPTG, GoldBio; St Louis, MO, USA) for 20 h at 16 °C. The cells were harvested by centrifugation at 4000 rpm (Fiberlite F9-6x1000 LEX Rotor; Thermo Lynx 6000; Waltham, MA, USA). The pellet was resuspended with lysis buffer (20 mM Tris-HCl, pH 8.0, 200 mM NaCl, and 10 mM dithiothreitol [DTT]) and disrupted by sonication. The lysate was centrifuged at 16,000 rpm (Fiberlite F21-8x50y Roter) for 30 min, and the supernatant fraction was incubated with glutathione Sepharose 4B resin (GE healthcare) in batch mode for 2 h. After extensive washing with lysis buffer, the beads were collected into a 10 ml column. On-column cleavage of the GST-tag was performed by the addition of homemade PreScission protease and gentle rotation at 4 °C overnight. The cleavage buffer consisted of 20 mM Tris-HCl, pH 8.0, 100 mM NaCl, and 10 mM DTT. The target proteins were eluted using the cleavage buffer, concentrated and loaded onto an anion exchange HiTrap Q HP column (GE Healthcare). Ints3-555-976 was eluted with a linear NaCl gradient and was further purified using a HiLoad 16/60 Superdex 200 gel filtration column (GE Healthcare) in buffer containing 20 mM Tris-HCl, pH 8.0, 150 mM NaCl, and 10 mM DTT. For other applications, GST-tagged proteins were used following partial purification and/or with the tag intact, as noted in the relevant sections below.

His-tagged proteins were expressed in the same fashion as GST-Ints3-555-976, except that 50 μg/ml kanamycin was used in the LB medium. The harvested cell pellet was resuspended in lysis buffer (20 mM Tris-HCl, pH 8.0, 400 mM NaCl, and 30 mM imidazole) and disrupted by sonication. The lysates were cleared by centrifugation at 16,000 rpm (Fiberlite F21-8x50y Roter) for 30 min and applied to HisPur Ni-NTA resin (Thermo; Waltham, MA, USA). After extensive washing with lysis buffer, the target proteins were eluted with 20 mM Tris-HCl, pH 8.0, 100 mM NaCl, and 400 mM imidazole and then supplemented with 10 mM DTT. Anion exchange chromatography (HiTrap Q HP column, GE Healthcare) and gel filtration (Superdex 200 Increase 10/300 Gl, GE Healthcare) were used sequentially to further purify the target proteins.

To purify the Ints3-Ints6 complex, Ints3-555-976 in pRSFDuet-1 and Ints6-807-887 in pGEX-6p-1 were used to cotransform BL21-CodonPlus (DE3)-RIPL cells. The complex was retrieved by GST-tagged Ints6-807-887 and purified following the GST-tagged protein purification protocol mentioned above. Ints3-555-976 can be replaced by Ints3-555-899 and the complex purified in the same way. For coexpression and interaction analysis by GST or MBP pulldowns, Ints3 truncations were cloned into pGEX-6p-1, while Ints6-807-887 was first ligated into a modified pMal-c2X vector (46) (New England Biolabs; Ipswich, MA, USA). The resulting construct has an AAAEF linker sequence and no protease site in between. This helps to alleviate the degradation problem observed with a long linker. MBP-Ints6-807-887 region was PCR-amplified again and cloned into pRSFDuet-1. Thus, the two proteins have GST or MBP-tag, and the two plasmids confer ampicillin or kanamycin resistance, respectively.

For *in vitro* GST pull-down, MBP-Ints6-807-887 inserted into pRSFDuet-1 was expressed by itself in BL21-CodonPlus (DE3)-RIPL cells. When OD_600_ of the cell culture reached 0.8–1.0, protein expression was induced by 0.25 mM IPTG for 20 h at 16 °C. The pellet was resuspended with lysis buffer (20 mM Tris-HCl, pH 8.0, 200 mM NaCl, and 10 mM dithiothreitol (DTT)) and disrupted by sonication. The lysates were cleared by centrifugation at 16,000 rpm (Fiberlite F21-8x50y Roter) for 30 min and applied to Amylose resin (New England Biolabs). After extensive washing with lysis buffer, the target proteins were eluted with 20 mM Tris-HCl, pH 8.0, 100 mM NaCl, and 10 mM maltose. Anion exchange chromatography (HiTrap Q HP column, GE Healthcare) was used to separate the MBP-Ints6-807-887 fusion protein from the MBP-tag. Purified proteins were flash-frozen in liquid nitrogen and stored at −80 °C.

### Protein crystallization, limited trypsin digestion, and edman sequencing

Purified Ints3-555-976 was concentrated to 10 mg/ml and subjected to crystallization screens by the sitting-drop vapor diffusion method at 16 °C. To set up trials for crystallization, the protein was mixed with precipitant at a ratio of 1:1 using the Phoenix protein crystallography robot (Art Robbins Instruments; Sunnyvale, CA, USA). Multiple commercial kits were screened, including those from Hampton Research (Aliso Viejo, CA, USA), Jena Bioscience (Jena, Germany), and NeXtal Tubes Protein Complex Suite (Hilden, Germany). Several initial hits were obtained, but could not be repeated.

Limited trypsin digestion was used to probe the disordered region in the Ints3-555-976 (Fig. S1). Protein (10 μg) was incubated with 0.1 μg trypsin (Sigma-Aldrich; St Louis, MO, USA) at room temperature for 30 s to 30 min. Reaction products were separated on SDS-PAGE gel. For Edman sequencing, the trypsin-cleaved protein sample was transferred to the Immobilon-FL PVDF membrane (Millipore; St Louis, MO, USA) after electrophoresis is complete. The PVDF membrane was stained with Coomassie blue, washed extensively with distilled water, and loaded onto a Shimadzu PPSQ-53A (Shimadzu; Kyoto, Japan) protein sequencer.

*In situ* proteolysis was used to facilitate crystallization. A trace amount of trypsin (Sigma-Aldrich; St Louis, MO, USA) was added at 1:1000 (mass ratio) and the sample was kept on ice from 4 h to overnight before crystallization screening. Trypsin treatment-generated fragments are very stable, and no further degradation was noticed in this process. Using this method, crystals could be repeated robustly in 0.2 M Magnesium Acetate Tetrahydrate, 13% PEG 3350. Crystals were transferred to cryo solutions containing 25% glycerol or 25% PEG400 before being flash-frozen in liquid nitrogen. For SelenoMethionine protein production, SelenoMethionine base medium, nutrient mix, and SelenoMethionine solution (250x) from Molecular Dimensions (Maumee, OH, USA) were used and the accompanying protocol was followed. Selenomethionine-containing protein crystals were grown under the same conditions.

### Structure determination

Both native and SeMet data sets were collected at The Northeastern Collaborative Access Team (NE-CAT) beamline 24-ID-C and 24-ID-E at the peak wavelength for Selenium 0.979 Å. Data were processed either with the automated NE-CAT RAPD server, which mainly uses XDS (47) or manually by using HKL2000 (48). The Ints3-555-976 structure was solved by SAD. Four SeMet data sets were merged to boost the Se-signal and increase the redundancy of the data (27). According to Phenix Xtriage (49), the measurability of anomalous signal slightly extended from 4.7–5.1 Å for individual data set to 4.3 Å for the merged data set. The redundancy and the low-resolution anomalous signal also got improved, and this finally yielded an interpretable electron density map (50). Selenium positions were found by Phenix AutoSol (Figure of merit: 0.36; Number of sites: 25), and the initial model was built by Phenix AutoBuild (49), incorporating native data at a higher resolution. This initial model was partial and had broken chains. Manual model building was performed using Coot (51), and the structure was refined with Phenix and Refmac (52, 53). A sharpened map was produced by CCP4i2 Refmac, which facilitates model rebuilding and does not affect model refinement. Secondary structure restraints and noncrystallographic twofold symmetry restraints were used throughout the refinement. The final round of refinement was performed in Phenix, and TLS refinement was added with each chain as a single group. The final model was refined to 3.11 Å with *R*_work_ and *R*_free_ of 0.204 and 0.263, respectively. Data scaling, refinement, and validation statistics are listed in Table S1. All figures were made using PyMOL (The PyMOL Molecular Graphics System, Version 1.7.4 Schrödinger, LLC.). The coordinates and structure factors have been deposited in the RCSB Protein Data Bank (PDB entry 6WLG)

### Model quality

Of the 14 methionine residues, 13 lie in the anomalous map peak contoured at 3.0 σ and one lies close to a peak (Fig. S3*A*). The N-terminal part of the structure (helices 1b–4b) appears to have more freedom in the crystal lattice and have many loop regions missing. Nevertheless, a construct without this region expressed poorly, suggesting an important role in the folding of the Ints3-555-976. We are not fully confident in the amino acid registration of helices 1b and 2b, and possible translocation along the helix axis exists. However, this is the best model having a lower *R*_free_ and clash score.

Electron density for helices 5a–10a (residues 719–898) is continuous and shows the best quality of the entire molecule (Fig. S3*B*). All the functionally key residues we have identified in this manuscript are located in this region. Helices 10b–11b are also well defined and have no gap. We deduce that trypsin treatment cleaved a disordered 20-amino-acid internal loop between helix 10a and 10b, which may hinder crystal packing (Fig. S1). Supporting this idea, both the recovered crystals and the overnight digested sample showed two bands in the SDS-PAGE gel. One band starts from the N-terminal end of the designed construct (proximately equivalent to Ints3-555-899), and the other band begins with residue 915 (Fig. 1*B* and Fig. S2, *B–C*). The latter one (presumably 915–976, 62 amino acids) is smaller than the 81-amino-acid construct Ints6-807-887 (Fig. S2*C*). From the electron density, N898 is the last residue that could be modeled after helix 10a, and Q917 is the first residue that could be modeled before helix 10b (Fig. 1*C*). The C-terminal residues up to E975 could also be reliably modeled. In summary, band size, Edman sequencing, and model building match with each other and support that loop connecting helix 10a–10b is cleaved.

### Light scattering analysis

Dynamic and static light scattering measurements were performed using a DynaPro NanoStar instrument (Wyatt Technology; Santa Barbara, CA, USA). Data were collected and analyzed with DYNAMICS v7 (7.9.0.5) software. Purified protein was syringe filtered through a 0.02 μm Whatman Anatop filter prior to measurements in 20 mM Tris-HCl, pH 8.0, 150 mM NaCl, and 2 mM DTT at similar protein concentrations. A 2 μl quartz cuvette was used for static light scattering (SLS), and 4 μl cyclic olefin copolymer (Wyatt) cuvettes were used for dynamic light scattering (DLS) temperature ramp experiments. Protein molecular weights were determined from multiple SLS measurements of the absolute average scattering intensity. DLS temperature ramps were done at 1 °C/min starting from 20 °C until the protein unfolded. Aggregation on-set temperature (T_onset_) was calculated using the linear fit module in DYNAMICS v7 from the change in overall hydrodynamic radius as temperature increased.

### Gel filtration analysis

To compare the gel filtration profiles of wild-type and mutant Ints3-555-976 proteins, 100 μl sample of approximately the same concentration (approximately 2 mg/ml) was injected into a Superdex 200 Increase 10/300 Gl column (GE Healthcare) with a flow rate of 0.5 ml/min. The injection loop and running buffer were also kept the same. For Figure 2*G*, The AKTA FPLC system (GE Healthcare) was used, and for Fig. S5*D*, the BioLogic DuoFlow system (Biorad; Hercules, CA, USA) was used.

### *In vitro* GST pull-down

To test the interactions between various Ints3 truncations or mutants with Ints6, GST-tagged Ints3 proteins were first captured onto 20 μl glutathione Sepharose 4B resin (5–10 mg/ml binding capacity) from an appropriate amount of BL21 lysates. Then, beads with bound GST-Ints3 proteins were incubated with approximately 15 μg purified MBP-Ints6-807-887 in binding buffer (20 mM Tris-HCl, pH 8.0, 300 mM NaCl, 0.1% Nonidet P-40, 5% glycerol, 2 mM DTT, and 0.4 mM phenylmethanesulfonyl fluoride (PMSF)) for 2 h at 4 °C. Beads were washed with binding buffer four times and boiled with 20 μl sample buffer. Sample (15 μl) was used for Coomassie blue staining, and the remaining 5 μl was used to detect the bound MBP-Ints6-807-887 by western blotting with anti-MBP.

### Electrophoretic mobility shift assays (EMSA)

Oligonucleotide dT30 (30 mer consecutive thymines) and rU30 (30 mer consecutive uracils) were purchased from Integrated DNA Technologies (IDT). The oligonucleotide was 5’ labeled with [γ-^32^P] ATP using T4 Polynucleotide Kinase (New England Biolabs). Unincorporated radionucleotides were removed by Oligo Clean & Concentrator Kit (Zymo Research; Irvine, CA, USA). The labeled ssDNA or ssRNA was stored at –20 °C until used.

For EMSA, increasing concentrations of the Ints3 C-terminal domain protein were incubated with labeled dT30 or rU30 (about 0.075 pmol, that is 7.5 nM in each reaction) in buffer (20 mM Hepes, pH 7.4, 100 mM NaCl, 1 mM MgCl_2_, 1 mg/ml bovine serum albumin, 2 mM DTT, and 5% glycerol). The binding reactions were kept on ice for 30 min in a 10 μl total volume. After incubation, loading dye was added and samples were loaded onto a prerun native 5% polyacrylamide gel (acrylamide/bisacrylamide 29:1) in 1X TBE buffer. Gels were run at 6 V/cm for 1.5 h. Gels were dried and exposed to a storage phosphor screen and the image visualized using a Storm 840 phosphor-imager. For quantification, band intensities were determined using the ImageJ program (NIH). The fraction of nucleic acids bound was calculated from the band intensities using the expression: bound/(bound+unbound). The fraction bound was plotted versus the protein concentration.

### Antibodies and immunoprecipitation

The following antibodies were used in this work: anti-His (Santa Cruz, sc-8036; Santa Cruz, CA, USA), anti-Flag (Sigma-Aldrich, F3165; St Louis, MO, USA), anti-MBP (Cell Signaling #2396; Danvers, MA, USA), anti-V5 (Invitrogen, R960–25; Carlsbad, CA, USA), anti-RNA Pol II (Santa Cruz, sc-17798), anti-Ints3 (Bethyl, A302–051A; Montgomery, TX, USA), anti-SSB1 (Bethyl, A301–938A), anti-β-actin (Santa Cruz, sc-47778), anti-GAPDH (Santa Cruz, sc-47724).

For immunoprecipitation, cells were disrupted in lysis buffer (20 mM Hepes, pH 7.9, 300 mM NaCl, 0.4% Nonidet P-40, 5% glycerol, 2 mM DTT, and 0.4 mM PMSF). Anti-Flag M2 Affinity Gel (Sigma-Aldrich) was used for immunoprecipitation of Flag-tagged proteins, and V5 epitope tag antibody agarose conjugate (Novus Biologicals; Littleton, CO, USA) was used for V5-tagged proteins. After incubation with antibody conjugates for 5 h, beads were washed with lysis buffer five times and 5 min each time. The last wash was performed with the lysis buffer containing 0.02% Nonidet P-40. Precipitated proteins were resolved by SDS-PAGE and detected using the indicated antibodies.

### Mammalian cell culture, shRNA-mediated gene knockdown, and rescue experiment

HEK293 T cells were cultured at 37 °C with 5% CO_2_ in Dulbecco’s modified Eagle’s medium supplemented with 10% fetal bovine serum (Omega Scientific, Tarzana, CA), 1X Penicillin-Streptomycin solution (GenDEPOT). For transient transfection, polyethylenimine (PEI)-based method was used (54). Lentivirus was produced by cotransfecting pLKO.1-shRNA, psPAX2, and pMD2.G plasmids into HEK293 T cells. The shRNA plasmid used was from the MISSION shRNA library (TRCN0000074393) with the hairpin sequence: CCAGTGTGAAATGGGCATCTA, which targets the 3UTR region. For the rescue experiment, HEK293 T cells were first transduced and selected with 1 μg/ml puromycin. Then cells with successful Ints3 knockdown were transfected with wild-type or mutant Ints3 plasmids, and SSB1 protein expression level was detected by western blotting.

### Circular dichroism

Protein secondary structural elements were assessed with a Jasco J-815 Circular Dichroism Spectrometer. CD spectra were collected at 10 °C from 260 to 190 nm with a data pitch of 1 nm, bandwidth of 1 nm, data integration time of 2 s, and scanning speed of 50 nm/min in a 1 mm quartz cuvette (Hellma). Proteins were diluted to 10 μM and measured in 20 mM Tris pH 8.0, 150 mM NaCl, and 2 mM DTT.

## Data availability

The coordinates and structure factors for the Ints3 C-terminal domain were deposited in the Protein Data Bank under accession number: 6WLG. All data are contained within the article and the supplementary material.

## Conflict of interest

The authors declare that they have no conflicts of interest with the contents of this article.
