# Supplementary material for: Structural basis for multifunctional roles of human Ints3 C-terminal domain
Source: J Biol Chem. 2020 Dec 3;296:100112. doi: 10.1074/jbc.RA120.016393 (PMC7948952; doi:10.1074/jbc.RA120.016393)
Supplement: Figures and Table [file mmc1.pdf]

Supplementary Figure S1

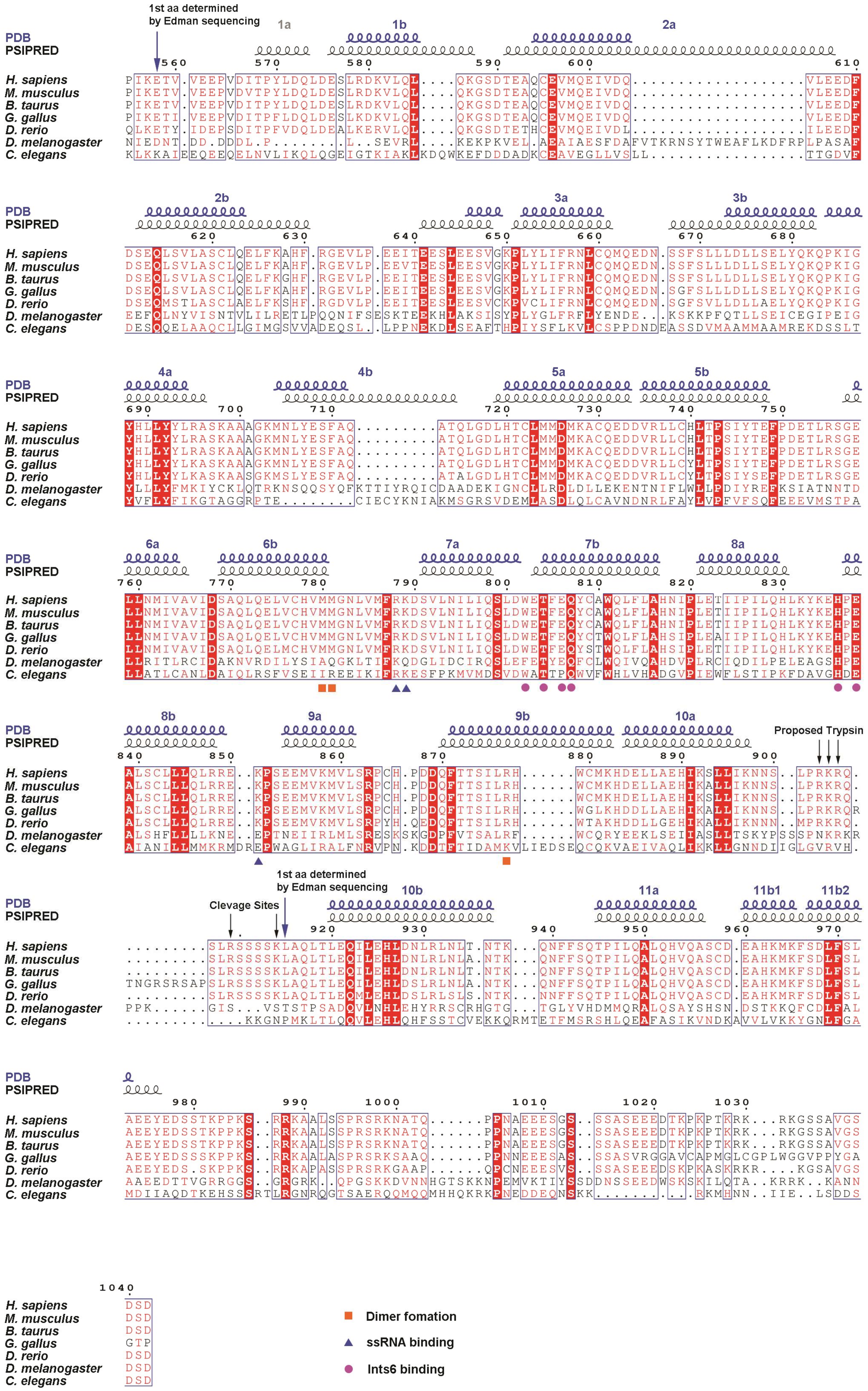

Supplementary Figure S1. Ints3 C-terminal domain multiple sequence alignments. Ints3 C-terminal domain amino acid sequences from human (Uniprot Q68E01), mouse (Uniprot Q7TPD0), bovine (Uniprot E1BN70), chicken (Uniprot A0A3Q2TSH0), zebrafish (Uniprot Q1LXC9), fruit fly (Uniprot Q7PLS8) and C. elegance (W6RQY9) were aligned using Clustal Omega (1) and displayed with ESPript 3 (2). The secondary structure of human Ints3 observed in our model and predicted by PSIPRED (3) is displayed on top of the sequence in blue and black, respectively. Proposed trypsin cleavage sites during *in situ* proteolysis are labeled with black downward arrows. For the trypsin treatment generated two fragments (Band II and III in Fig. S2D), the first amino acid determined by Edman degradation is pointed by a blue downward arrow. Key residues later found to be important for dimer formation, single-stranded RNA (ssRNA) binding, and Ints6 binding are labeled with orange squares, blue triangles, and magenta dots, respectively.

Supplementary Figure S2

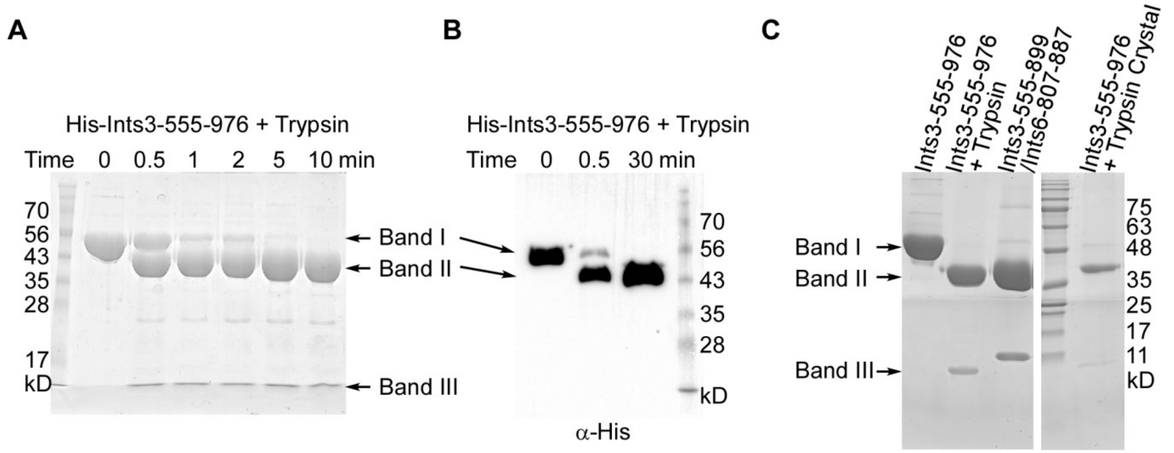

Supplementary Figure S2. Limited trypsin digestion and *in situ* proteolysis. (A) Limited trypsin digestion reveals a stable fragment (Band II) slightly lower than the untreated Ints3-555-976 (Band I). Although not resolved, a much smaller fragment (Band III) was also generated, which is clearly observed in subfigure (C). (B) Western blotting against N-terminal His-tag demonstrates that Band II corresponds to the N-terminal part of the construct and has an uncleaved His-tag. (C) *In situ* proteolysis by a trace amount of trypsin (1:1000 w/w) facilitated crystal growth. Both protein samples treated in this manner and harvested crystals show Band II/III in SDS-PAGE gel. Notably, Band II has a similar size with Ints3-555-899, whereas Band III appears smaller than Ints6-807-887 (81 amino acids).

Supplementary Figure S3

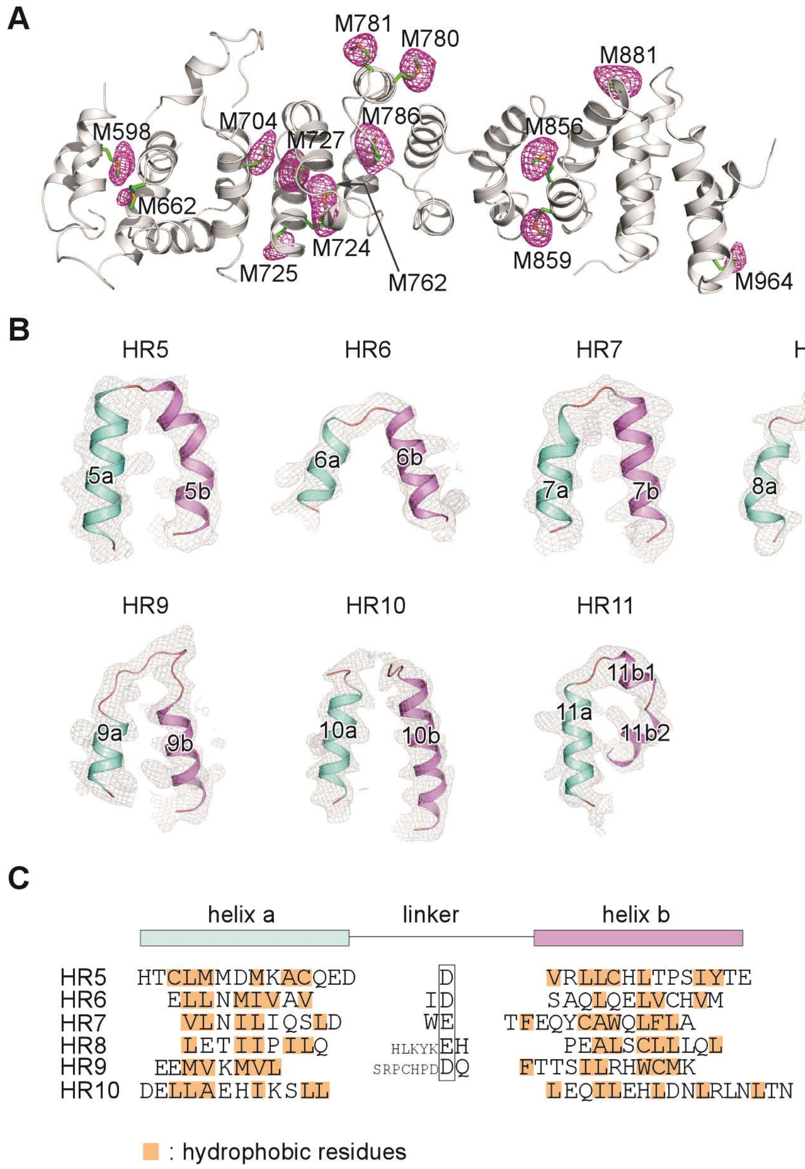

Supplementary Figure S3. Electron densities of Ints3-555-976 crystal structure. (A) Anomalous map contoured at 3.0 σ (magenta mesh). Of the 14 methionine residues, 13 fit the anomalous map perfectly. One of them, M662, is close to a peak. HR1-4 has three methionine residues that can be used as anchoring points in the sidechain assignment, while HR5-11 has 11 methionines. (B) Helix-loop-helix structure of the individual HEAT repeat (HR5-11). 2mFo-DFc map contoured at 1.2 σ is shown as mesh surrounding each HEAT repeat. Sidechain electron density in the helices is clearly visible, which guarantees a decent model quality for the HR5-11 region. (C) The HEAT repeat motif is highly degenerate and a consensus sequence is hard to find. However, the helices are amphiphilic and

hydrophobic residues form intra- and inter-repeat interactions to bury their hydrophobic surfaces inside. Aspartic acid residues are often found in the turn region.

Supplementary Figure S4

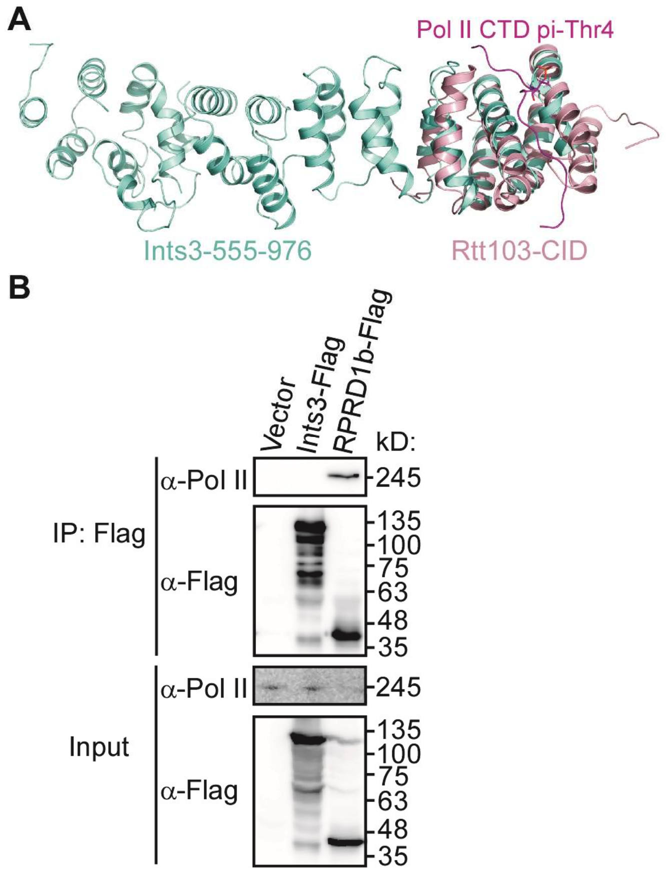

Supplementary Figure S4. Structural similarity with RNA polymerase II C-terminal domain (CTD)-interacting domain (CID). (A) Structural alignment with Rtt103-CID complexed with Thr4 phosphorylated Pol II CTD (PDB ID 5LVF). Rtt103-CID and RPRD1b-CID are among the top structural homologues identified by the Dali server (4). (B) Potential interaction between Ints3 and RNA polymerase II CTD was tested using a coimmunoprecipitation assay in HEK293T cells. Cells were transfected with Flag-tagged Ints3 or RPRD1b and immunoprecipitated using Flag M2 agarose. The presence of endogenous RNA polymerase II in the precipitated material was examined by Western blotting. RPRD1b (5), but not Ints3, displays strong binding to Pol II.

Supplementary Figure S5

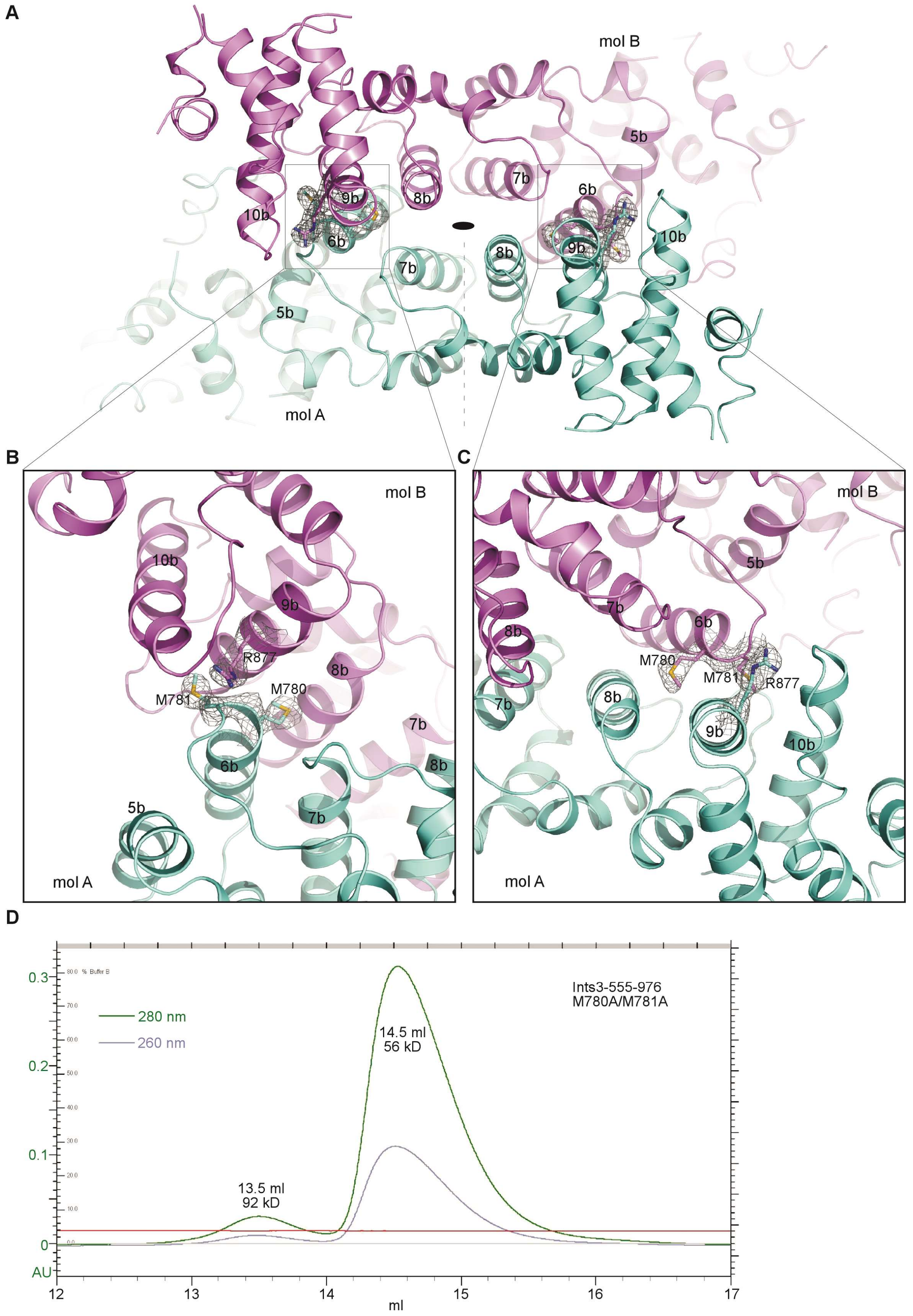

Supplementary Figure S5. Detailed view of the dimer interface. (A) The dimer interface is composed of helices 6b, 7b, 8b, 9b from both molecule A and B. Due to the two-fold rotational symmetry, this interface is centered around two half-sites (gray boxes). The enlarged views are in (B) and (C), rotated to better depict the details. (B) In one half-site, helix 6b (mol A) makes extensive contacts with 8b/9b (mol B). (C) Correspondingly, in the other half-site, helix 6b (mol B) makes extensive contacts with 8b/9b (mol A). 2mFo-DFc type electron density map (with B-factor sharpening, produced by CCP4i2 Refmac) around 3 critical residues and contoured at 1.5 σ are shown as mesh. (D) M780A/M781A mutant protein still retains some dimeric species, which could be resolved to a minor 92 kD peak and a major 56 kD peak, using an independently calibrated gel filtration column.

Supplementary Figure S6

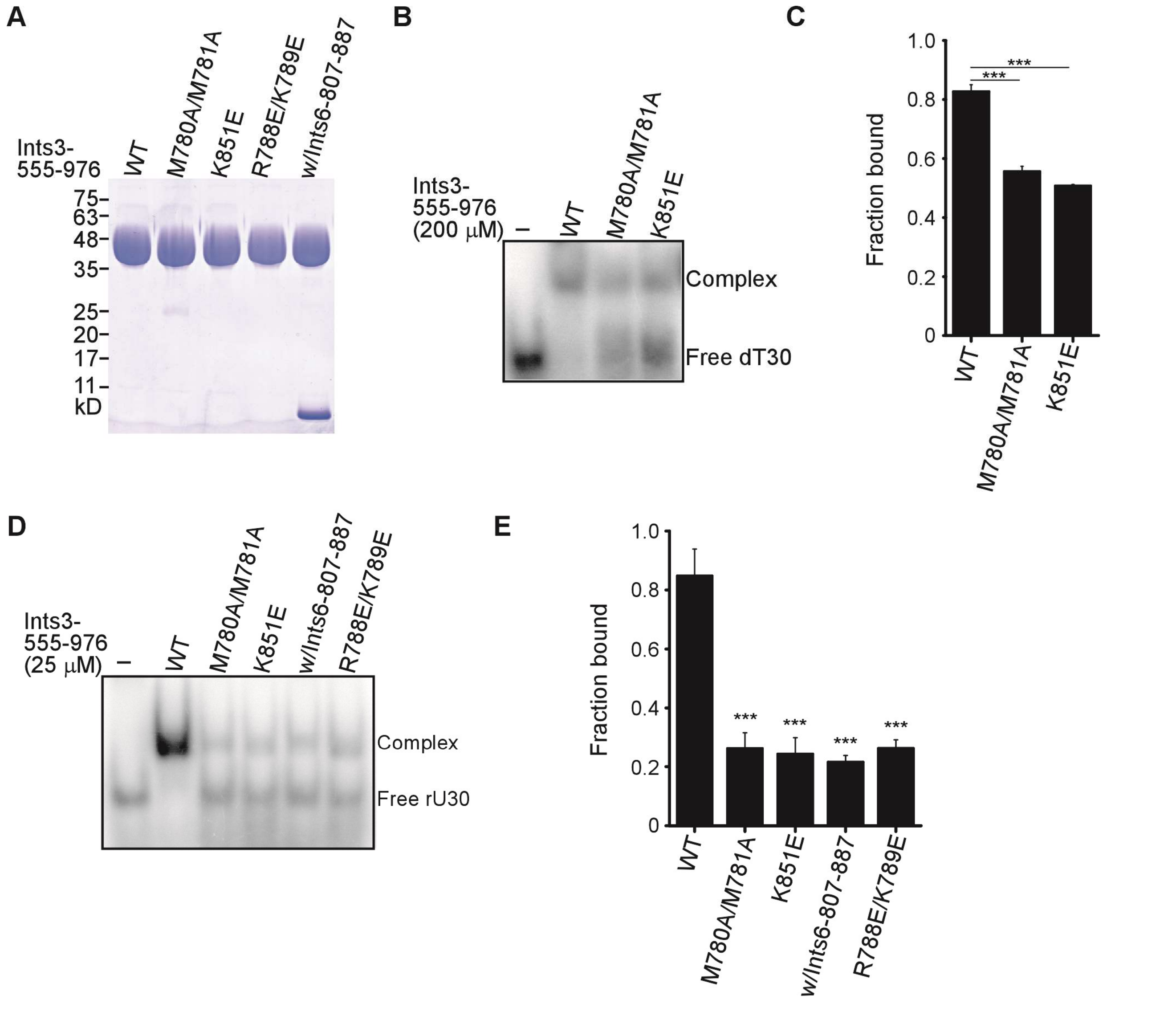

Supplementary Figure S6. Nucleic acid binding by the Ints3 C-terminal domain. (A) Purified proteins used in the EMSA experiments. w/Ints6-807-887 denote Ints3-555-976 complexed with Ints6. (B) Binding of the Ints3 C-terminal domain with dT30. Protein concentration was fixed at 200 µM. A representative image of EMSA is shown. (C) Quantification of (B) from 3 independent repeats. Compared with wildtype protein, mutants have significantly reduced dT30 binding activity. Error bars represent standard deviation. Significance was tested by unpaired Student's *t*-test: ***, $p<0.001$. (D) Binding of the Ints3 C-terminal domain with rU30. Protein concentration was fixed at 25 µM. A representative image of EMSA is shown. (E) Quantification of (D) from 3 independent repeats.

Compared with wildtype protein, mutants and Ints6 complex have significantly reduced rU30 binding activity (***, $p<0.001$).

Supplementary Figure S7

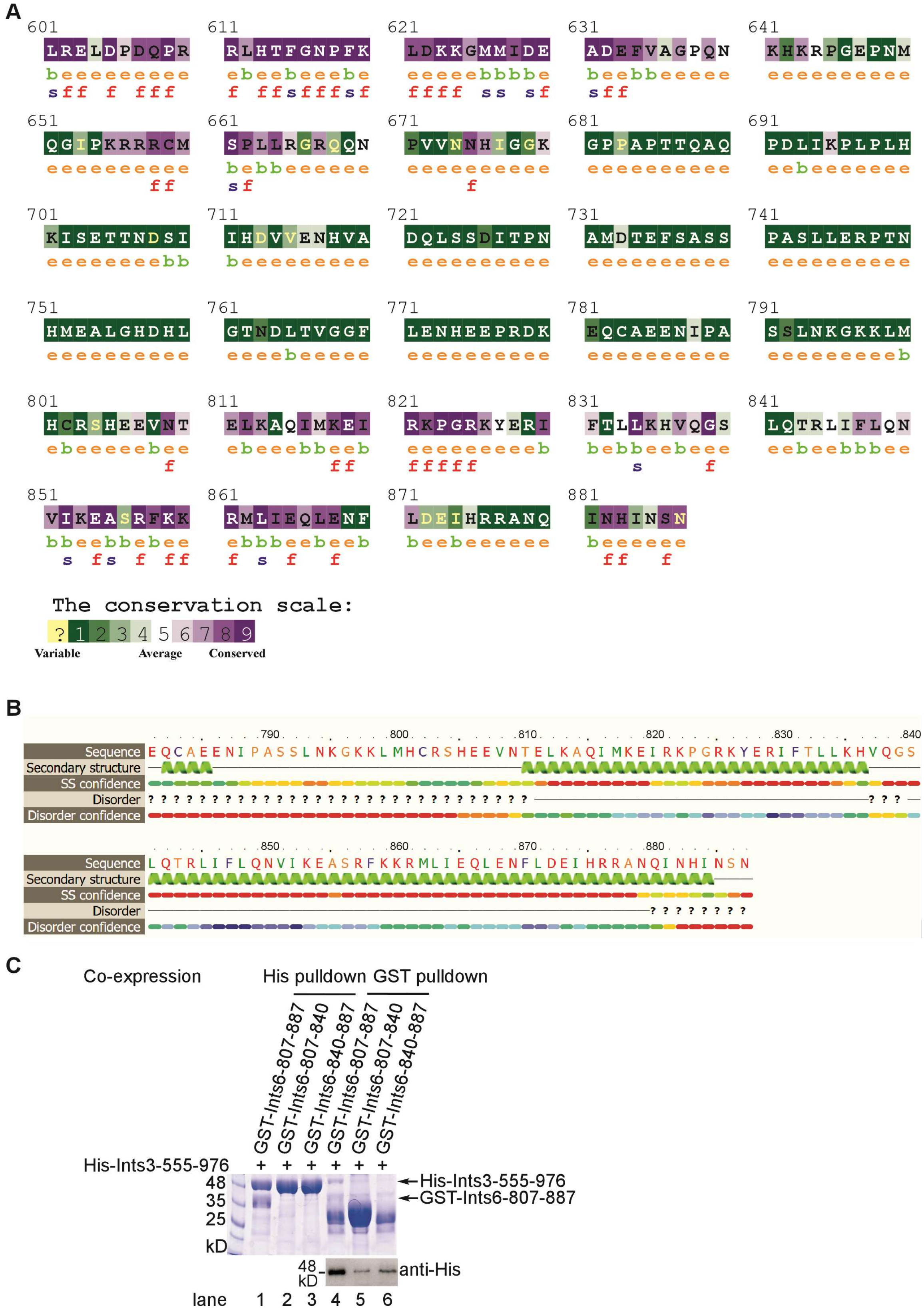

Supplementary Figure S7. Conservation and secondary structure analysis of the Ints3 binding region within Ints6. (A) Ints6 contains a conserved N-terminal domain (approximately residues 1-680) and a conserved C-terminus (residues 807-887). Linking them is a highly variable 120 amino acid segment (6). A previous report implicated residues 747-887 are vital for Ints3 interaction (7), which included some variable and possibly unnecessary region (residues 747-806). (B) Secondary structure prediction showed that the Ints6 C-terminus contains two segments of α-helix beginning from amino acid 810 (8), echoing the conservation analysis. (C) GST-tagged Ints6 C-terminus harboring either one or both helices were co-expressed with His-tagged Ints3-555-976 in *E. coli* BL21. Bacterial lysates were incubated with either Glutathione Sepharose beads or HisPur Ni-NTA resin, washed extensively, and resolved by SDS-PAGE. Ints6-807-887 appears to be the smallest fragment that could form a stable complex with Ints3.

Supplementary Figure S8

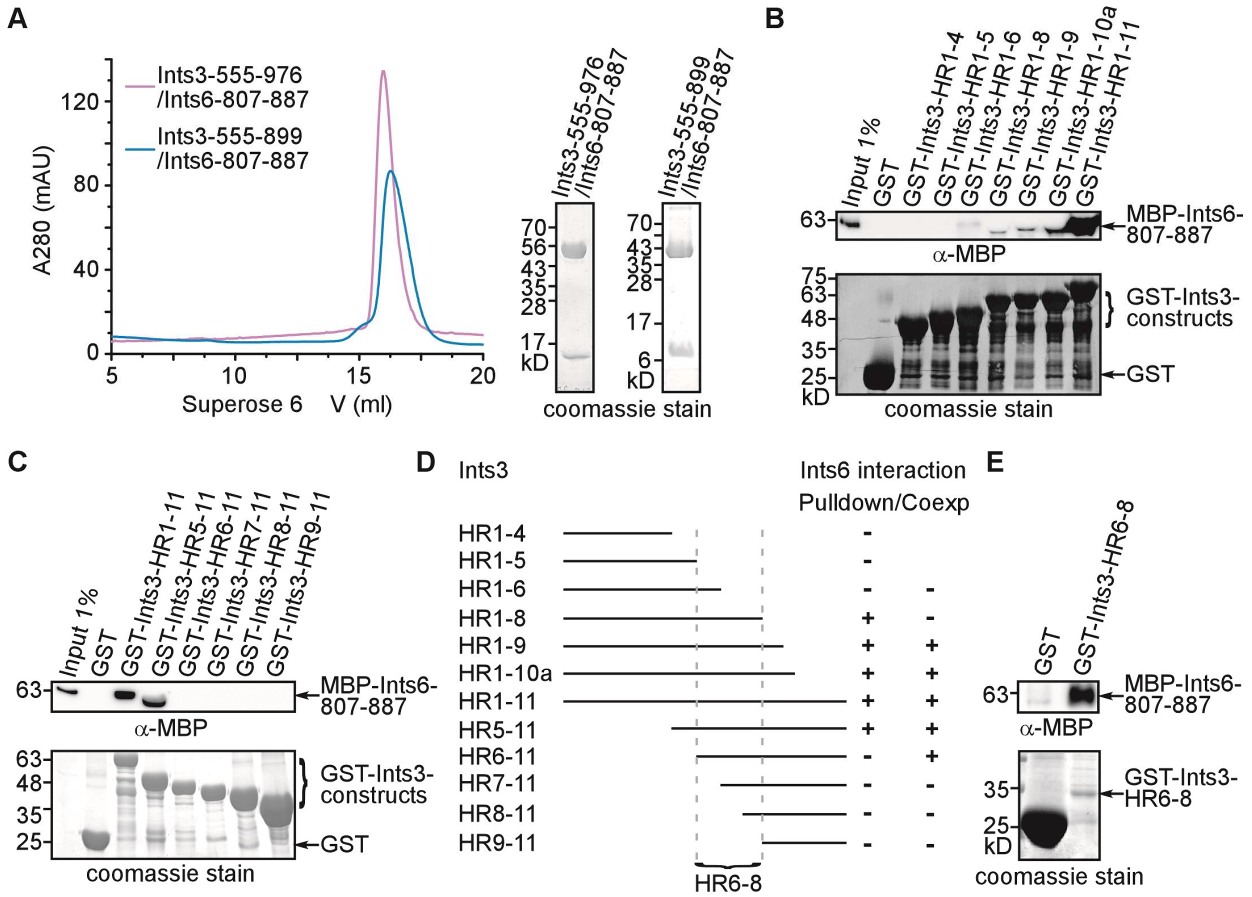

Supplementary Figure S8. Mapping of the region required for Ints6 binding. (A) When co-expressed, Ints3-555-976 and Ints3-555-899 are able to form stable complexes with Ints6-807-887, which could be purified to homogeneity and exist as a single peak in gel filtration (Superose 6 Increase 10/300 GL). (B) Pulldown of Ints6-807-887 by GST-tagged Ints3 C-terminal domain proteins. One or more HEAT repeats from the C-terminus were deleted. The truncated forms of GST-tagged Ints3 were first immobilized on glutathione Sepharose beads, and then incubated with a purified MBP-tagged Ints6-807-887 protein. After extensive washing, MBP-Ints6-807-887 bound to Ints3 was detected using an MBP antibody. Representative result from at least 3 repeats is shown. (C) Pulldown of Ints6-807-887 by GST-tagged Ints3 C-terminal domain proteins, which have one or more N-terminal HEAT repeats chopped off. The experiment was performed similarly as in (B). (D) Summary of the interaction data from *in vitro* GST pulldowns and co-expression studies (Coexp, Fig. S9). Fragments harboring HR6-8 showed positive Ints6 binding in at least one experimental method, suggesting this is the minimal region. (E) Albeit poor expression, GST-tagged Ints3 HR6-8 could pulldown Ints6 in the co-expression experiment.

Supplementary Figure S9

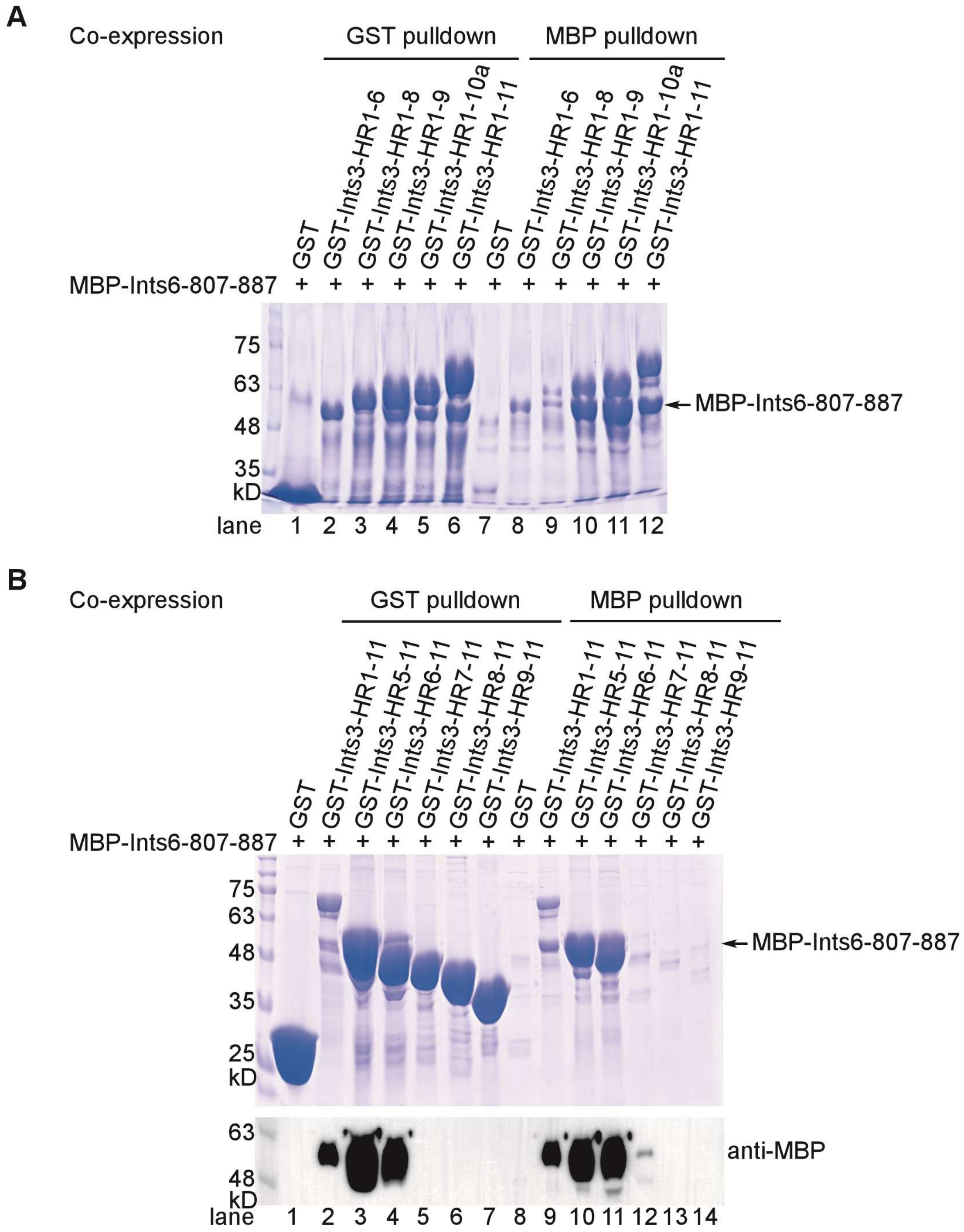

Supplementary Figure S9. Co-expression studies of the interaction between Ints3 C-terminal domain truncations and Ints6-807-887. (A) A series of GST-tagged Ints3 C-terminal domain

truncations from the C-terminus were co-expressed with MBP-tagged Ints6-807-887 in *E. coli* BL21. These truncations were designed following the boundaries of HEAT repeats. Bacterial lysates were incubated with either Glutathione Sepharose beads or Amylose resin, washed extensively, and resolved by SDS-PAGE. MBP-Ints6-807-887 was not stable by itself (lane 7). When complexed with an Ints3 fragment capable of binding it, MBP-Ints6-807-887 was stabilized and accumulated to a higher expression level (compare lanes 10-12 with lanes 8-9). Under these circumstances, GST-tagged Ints3 truncation protein and MBP-Ints6-807-887 are able to pull down each other (lanes 4-6 and lanes 10-12). Regarding the Ints6 binding ability, Ints3 HR1-9 is the smallest construct that is not affected by chopping the C-terminus. Representative result from at least 2 repeats is shown. (B) A series of GST-tagged Ints3 C-terminal domain truncations from the N-terminus were co-expressed with MBP-tagged Ints6-807-887 in *E. coli* BL21. Similar to the untruncated C-terminal domain, Ints3 HR5-11 and Ints3 HR-6-11 were able to form complexes with and stabilize MBP-Ints6-807-887 (lanes 2-4, and lanes 9-11). For Ints3 fragments that did not interact with Ints6, MBP-Ints6-807-887 is unstable and expressed poorly (compare lanes 12-14 with lanes 9-11). Due to the proximity of some bands, the presence of MBP-Ints6-807-887 was also confirmed by Western blotting against the MBP-tag. Regarding the Ints6 binding ability, Ints3 HR6-11 is the smallest construct that is not affected by chopping the N-terminus. Representative result from at least 2 repeats is shown.

Supplementary Figure S10

A

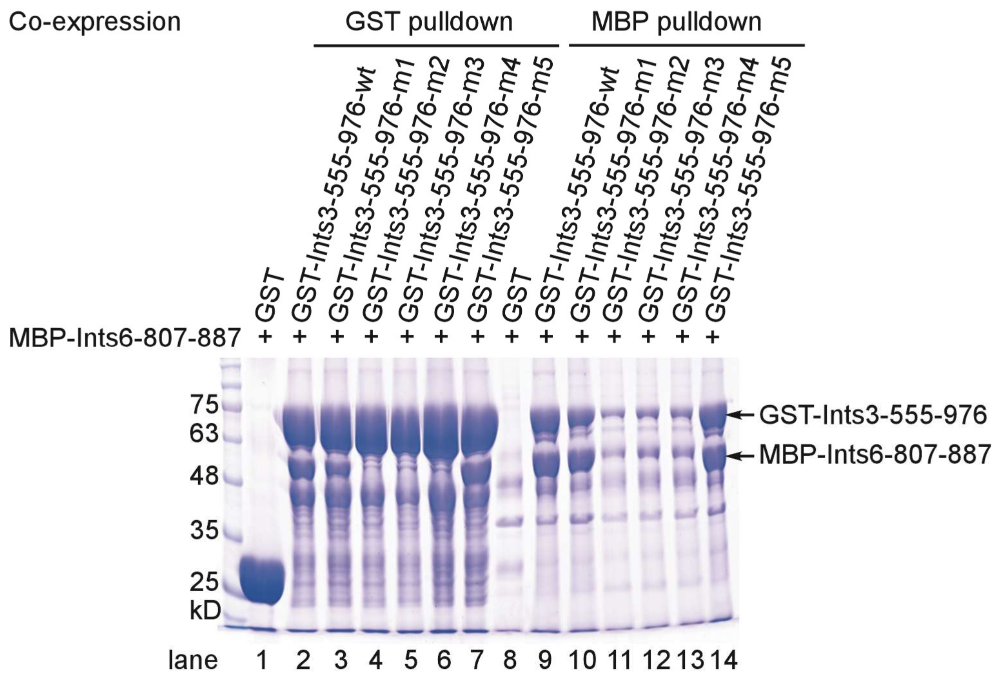

B

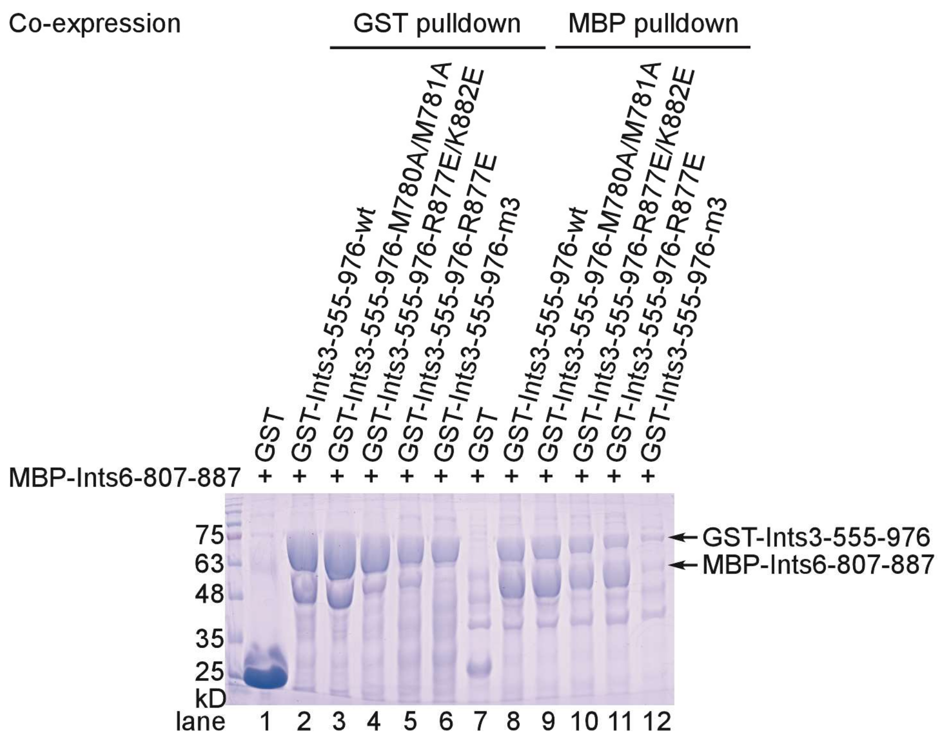

Supplementary Figure S10. Co-expression studies of the interaction between Ints3 C-terminal domain mutants and Ints6-807-887. (A) A series of GST-tagged Ints3 C-terminal domain mutants (m1-m5) were co-expressed with MBP-tagged Ints6-807-887 in *E. coli* BL21. Similar to wildtype C-terminal domain, m1 and m5 were able to form complexes with and stabilize MBP-Ints6-807-887 (lanes 2,3,7 and lanes 9,10,14). GST-tagged m2-m4 failed to pulldown Ints6 (lanes 4-6), and meanwhile, MBP-Ints6-807-887 is poorly expressed and complex formation reduced (compare lanes 11-13 with lanes 9,10,14). Representative result from at least 2 repeats is shown. (B) A series of GST-tagged Ints3 C-terminal domain monomeric mutants were co-expressed with MBP-tagged Ints6-807-887 in *E. coli* BL21. Similar to the wildtype C-terminal domain, monomeric mutants M780A/M781A, R877E/K882E, and R877E were able to form complexes with and stabilize MBP-Ints6-807-887. These complexes can be retrieved by either GST or MBP pulldown (lanes 2-5 and lanes 8-11). Mutant m3 was used as a negative control. Thus dimerization is not required for Ints6 interaction. Representative result from at least 2 repeats is shown.

Supplementary Figure S11

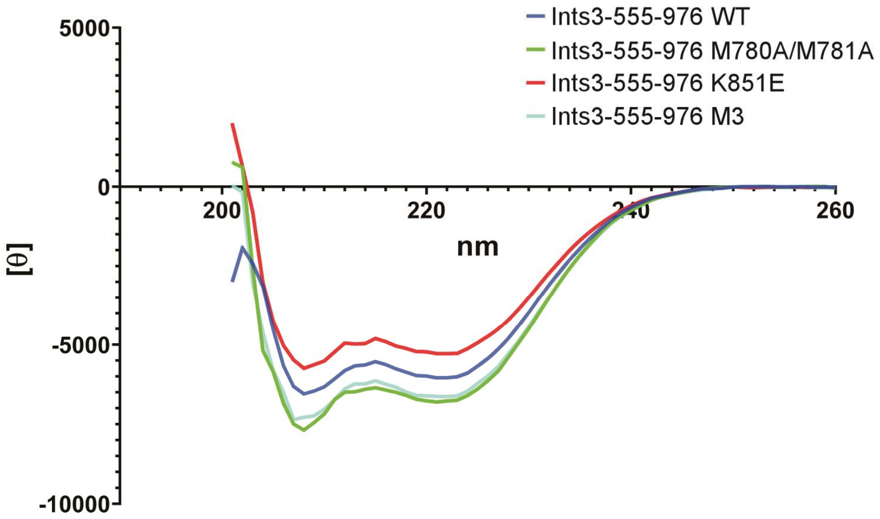

Figure S11. Analysis of the secondary structure of wildtype and selected Ints3 C-terminal domain mutants using Circular Dichroism (CD). All four proteins have similar α-helical secondary structure, indicating no large structural changes caused by mutation.

Supplementary References

1. Madeira, F., Park, Y.M., Lee, J., Buso, N., Gur, T., Madhusoodanan, N., Basutkar, P., Tivey, A.R.N., Potter, S.C., Finn, R.D. *et al.* (2019) The EMBL-EBI search and sequence analysis tools APIs in 2019. *Nucleic acids research*.
2. Robert, X. and Gouet, P. (2014) Deciphering key features in protein structures with the new ENDscript server. *Nucleic acids research*.
3. Buchan, D.W.A. and Jones, D.T. (2019) The PSIPRED Protein Analysis Workbench: 20 years on. *Nucleic acids research*, **47**, W402-W407.
4. Holm, L. (2019) Benchmarking fold detection by DaliLite v.5. *Bioinformatics*, **35**, 5326-5327.
5. Ni, Z., Xu, C., Guo, X., Hunter, G.O., Kuznetsova, O.V., Tempel, W., Marcon, E., Zhong, G., Guo, H., Kuo, W.W. *et al.* (2014) RPRD1A and RPRD1B are human RNA polymerase II C-terminal domain scaffolds for Ser5 dephosphorylation. *Nature structural & molecular biology*, **21**, 686-695.
6. Ashkenazy, H., Abadi, S., Martz, E., Chay, O., Mayrose, I., Pupko, T. and Ben-Tal, N. (2016) ConSurf 2016: an improved methodology to estimate and visualize evolutionary conservation in macromolecules. *Nucleic acids research*, **44**, W344-350.
7. Zhang, F., Ma, T. and Yu, X. (2013) A core hSSB1-INTS complex participates in the DNA damage response. *Journal of cell science*, **126**, 4850-4855.
8. Kelley, L.A., Mezulis, S., Yates, C.M., Wass, M.N. and Sternberg, M.J. (2015) The Phyre2 web portal for protein modeling, prediction and analysis. *Nature protocols*, **10**, 845-858.

Supplementary Table S1. Data scaling, refinement, and validation statistics

**Crystals**	Native #a3	SeMet #b1	SeMet #b2	SeMet #b3	SeMet #b7
**Data collection**					
Space group	*H3*	*H3*	*H3*	*H3*	*H3*
Cell dimension					
a,b,c(Å)	234.85, 234.85, 47.57	236.07, 236.07, 48.04	236.79, 236.79,48.23	235.64, 235.64, 48.00	235.47, 235.47, 47.58
α,β,γ(°)	90, 90, 120	90, 90, 120	90, 90, 120	90, 90, 120	90, 90, 120
Resolution (Å)	46.32-3.11(3.33-3.11)[a]	118.04-3.51(3.85-3.51)	118.40-3.48(3.81-3.48)	117.82-3.60(3.94-3.60)	117.73-3.20(3.42-3.20)
R_{merge}(%)	6.0(57.0)	8.9(94.1)	10.5(215.3)	11.2(97.3)	9.3(130.3)
$I/\sigma I$	14.6(2.5)	8.8(1.2)	11.4(0.7)	7.6(1.2)	12.3(1.0)
$CC_{1/2}$	0.998(0.896)	0.994(0.539)	0.999(0.366)	0.992(0.468)	0.998(0.560)
Completeness (%)	99.5(99.2)	97.3(96.7)	95.1(93.9)	97.4(96.9)	98.8(97.4)
Multiplicity	5.3(5.3)	2.9(2.9)	5.5(5.5)	2.9(2.9)	5.3(5.3)
**Refinement**					
Resolution (Å)	46.32-3.11				
Total No. reflection	17485				
R_{work}/R_{free}	0.2037/0.2633				
r.m.s.d. bonds (Å)/angles (°)	0.003/0.777				
Protein/Solvent atom count	5750/0				
Average B-factor/Overall (Å^2)	152.50				
Chain A (Å^2)	139.82				
Chain B (Å^2)	165.75				
**Ramachandran plot statistics**					
Most favorable	92.15%				
Additionally allowed	6.52%				
Disallowed	1.33%				

[a] Values in parentheses are for highest-resolution shell.
